# Supplementary material for: Heterotrimeric Gq proteins act as a switch for GRK5/6 selectivity underlying β-arrestin transducer bias
Source: Nat Commun. 2022 Jan 25;13:487. doi: 10.1038/s41467-022-28056-7 (PMC8789823; doi:10.1038/s41467-022-28056-7)

SUPPLEMENTARY INFORMATION

Heterotrimeric Gq proteins act as a switch for GRK5/6 selectivity underlying β-arrestin transducer bias

Kouki Kawakami[1,6], Masataka Yanagawa[2,6], Suzune Hiratsuka[1], Misaki Yoshida[1], Yuki Ono[1], Michio Hiroshima[2,3], Masahiro Ueda[3,4], Junken Aoki[5], Yasushi Sako[2*], Asuka Inoue[1*]

[1]*Molecular and Cellular Biochemistry, Graduate School of Pharmaceutical Sciences, Tohoku University, Sendai, Miyagi, 980-8578, Japan.*

[2]*Cellular Informatics Laboratory, RIKEN Cluster for Pioneering Research, 2-1 Hirosawa, Wako, Saitama 351-0198, Japan.*

[3]*Laboratory for Cell Signaling Dynamics, RIKEN BDR, 6-2-3, Furuedai, Suita, Osaka 565-0874, Japan.*

[4]*Laboratory of Single Molecule Biology, Graduate School of Frontier Biosciences, Osaka University, 1-3 Yamadaoka, Suita, Osaka 565- 0871, Japan.*

[5]*Graduate School of Pharmaceutical Sciences, The University of Tokyo, Tokyo, 113-0033, Japan.*

[6]*These authors contributed equally*

* To whom correspondence should be addressed. E-mail: sako@riken.jp and iaska@tohoku.ac.jp

Contents:

Supplementary Fig. 1 Genomic sequences of the GRK-deficient HEK293A cell lines.

Supplementary Fig. 2 Characterization of the GRK-deficient cell lines.

Supplementary Fig. 3 AT1R biased ligands and their GRK-subtype dependency.

Supplementary Fig. 4 Contribution of the individual GRKs to β-arrestin-recruitment responses.

Supplementary Fig. 5 GRK-dependent AT1R phosphorylation.

Supplementary Fig. 6 GRK-dependent AT1R internalization and β-arrestin endosomal translocation.

Supplementary Fig. 7 ERK phosphorylation and β-arrestin conformational change under the Gq-inactivated condition.

Supplementary Fig. 8 FLR-dependent β-arrestin functions.

Supplementary Fig. 9 AT1R phosphorylation-independent, tail-free core engagement of β-arrestin.

Supplementary Fig. 10. Dual-color SMT analysis of AT1R-GRK5 and AT1R-GRK2.

Supplementary Fig. 11 Single-molecule diffusion behaviors of AT1R and GRK5 molecules.

Supplementary Fig. 12 Apparent oligomer size of AT1R and GRK5 molecules.

Supplementary Fig. 13 Single-molecule behaviors of colocalized AT1R and GRK molecules.

Supplementary Fig. 14 Diffusion state fractions of AT1R and GRK5 molecules in ΔGq cells.

Supplementary Fig. 15 GRK recruitment to AT1R under the Gq-inactivated condition.

Supplementary Fig. 16 Effect of Gq downstream signaling on the GRK5/6 utilization.

Supplementary Fig. 17 GRK5/6 association to Gq and conformational change under the Gq-inactivated condition.

Supplementary Fig. 18 YM-triggered GRK-subtype selectivity switch in Gq- and non-Gq-coupled receptors.

Uncropped images of Western blot analysis

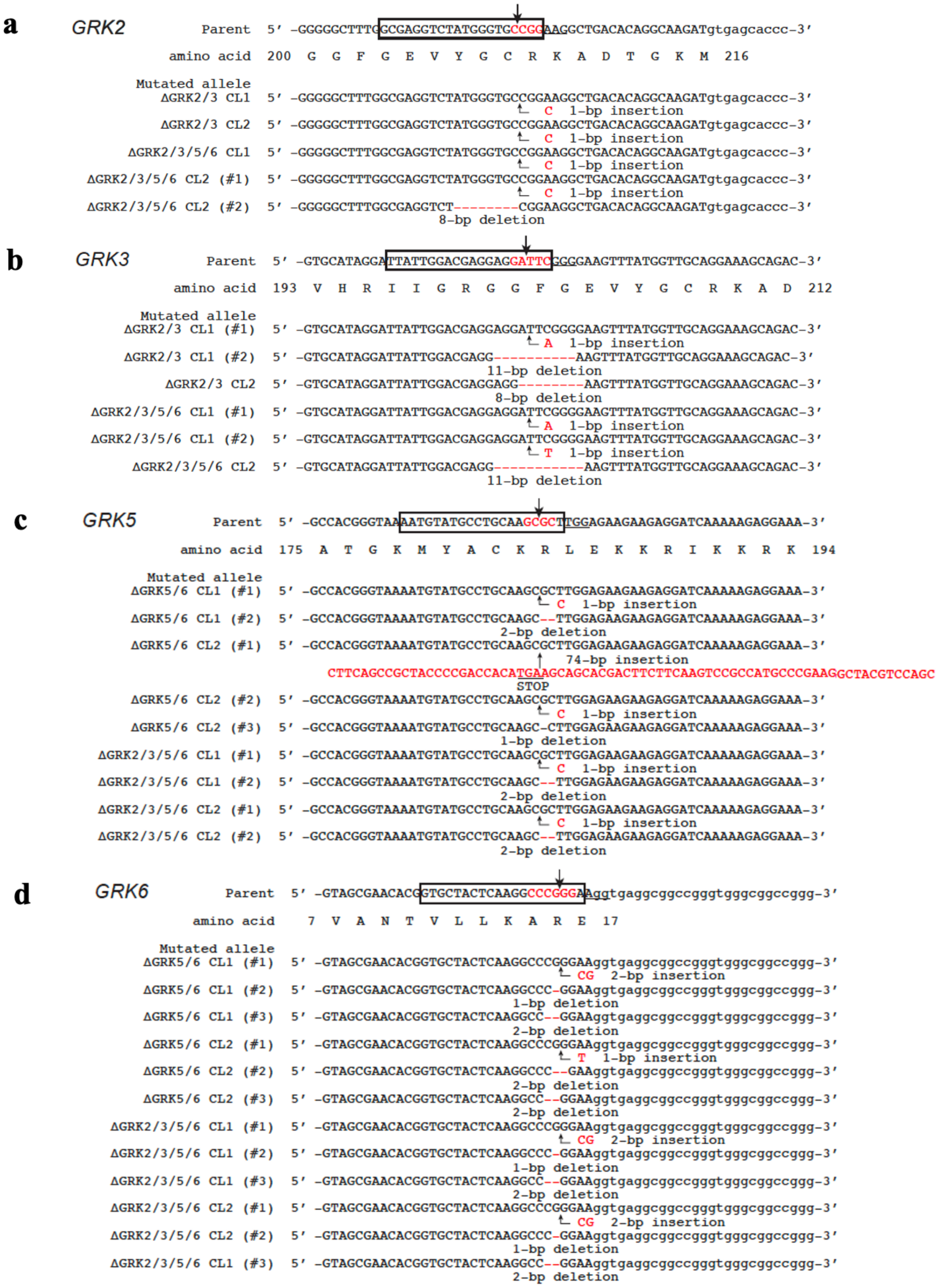

Supplementary Fig. 1 Genomic sequences of the GRK-deficient HEK293A cell lines.

a-d, sgRNA-target sequences of the individual clones were determined by a direct sequencing method or a TA-cloning method. sgRNA target sequences are boxed and the SpCas9 PAM sequences (NGG) are underlined. Arrows indicate a putative double-stranded break site. Restriction enzyme sites (Hap II (*GRK2*), Hinf I (*GRK3*), Hha I (*GRK5*) and Sma I (*GRK6*)) are highlighted in red.

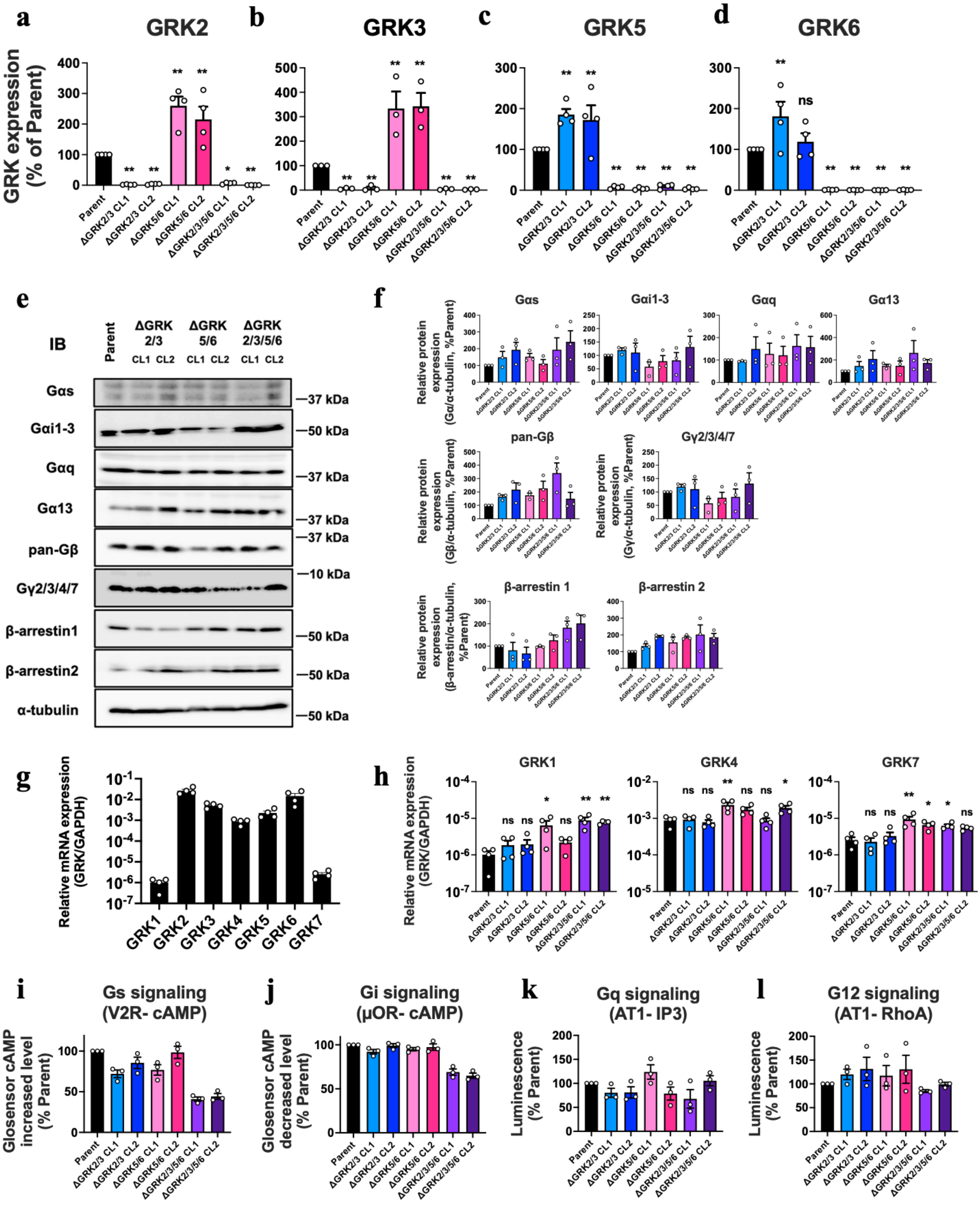

a
GRK2
GRK expression (% of Parent)
b
GRK3
c
GRK5
d
GRK6
Parent
ΔGRK2/3 CL1
ΔGRK2/3 CL2
ΔGRK5/6 CL1
ΔGRK5/6 CL2
ΔGRK2/3/5/6 CL1
ΔGRK2/3/5/6 CL2
e
IB
Parent
ΔGRK 2/3
ΔGRK 5/6
ΔGRK 2/3/5/6
CL1 CL2
Gαs
Gαi1-3
Gαq
Gα13
pan-Gβ
Gγ2/3/4/7
β-arrestin1
β-arrestin2
α-tubulin
37 kDa
50 kDa
10 kDa
f
Gαs
Gαi1-3
Gαq
Gα13
Relative protein expression (Gα/α-tubulin, %Parent)
pan-Gβ
Gγ2/3/4/7
Relative protein expression (Gβ/α-tubulin, %Parent)
Relative protein expression (Gγ/α-tubulin, %Parent)
β-arrestin 1
β-arrestin 2
Relative protein expression (β-arrestin/α-tubulin, %Parent)
g
Relative mRNA expression (GRK/GAPDH)
GRK1
GRK2
GRK3
GRK4
GRK5
GRK6
GRK7
h
GRK1
GRK4
GRK7
i
Gs signaling (V2R- cAMP)
Glosensor cAMP increased level (% Parent)
j
Gi signaling (μOR- cAMP)
Glosensor cAMP decreased level (% Parent)
k
Gq signaling (AT1- IP3)
Luminescence (% Parent)
l
G12 signaling (AT1- RhoA)

Supplementary Fig. 2 Characterization of the GRK-deficient cell lines.

a-d, Western blot analyses were performed by using lysates derived from wild-type (parent HEK293) and ΔGRK2/3, ΔGRK5/6 and ΔGRK2/3/5/6. Membranes were probed with the indicated antibodies against GRK2, 3, 5, 6 or α-tubulin. α-tubulin was used as a loading control. Quantification of chemiluminescent band intensity in the western blot analyses. For each sample, GRK expression levels were normalized to that of α-tubulin and shown relative to parental cells. **e, f**, Western blot analysis of the parent, ΔGRK2/3, ΔGRK5/6 and ΔGRK2/3/5/6 cells. Expression levels are shown as relative values to that in parental cells. α-tubulin was used as a loading control. **g,** Quantitative real-time PCR (qRT-PCR) analysis of the GRK family members in the parental cells. GRK mRNA copy numbers were normalized by that of *GAPDH* and relative expression levels were quantified by the ΔΔCT method. **h**, qRT-PCR analyses of the untargeted GRK subtypes in the parental and the GRK-deficient cell lines. **i-l**, Functional assessment of G-protein signaling in the GRK-deficient cell lines. Gs signaling and Gi signaling were measured by the GloSensor cAMP assay using V2R (100 nM AVP) and μOR (1 μM DAMGO in the presence of 10 μM forskolin), respectively. Gq signaling and G12 signaling were measured by the NanoBiT-IP_3 assay and the NanoBiT-RhoA assay, respectively, using AT1R (1 μM Ang II). For Gs signaling, cAMP response was normalized to forskolin (10 μM)-induced responses. For all cases, responses in the GRK-deficient cells were normalized to that of the parietal cells. In all panels, bars and error bars represent mean and SEM, respectively, of 3 (f, i, j, k, l) or 3-4 (b, d, h) independent experiments with each performed in duplicate. In Fig.2a, d, h, * and ** represent $P < 0.05$ and 0.01, respectively, with one-way ANOVA followed by the Dunnett's test for multiple comparison analysis (with reference to the Parent). ns, not significantly different between the groups. See Supplementary statistics data file for additional statistics and exact *P* values.

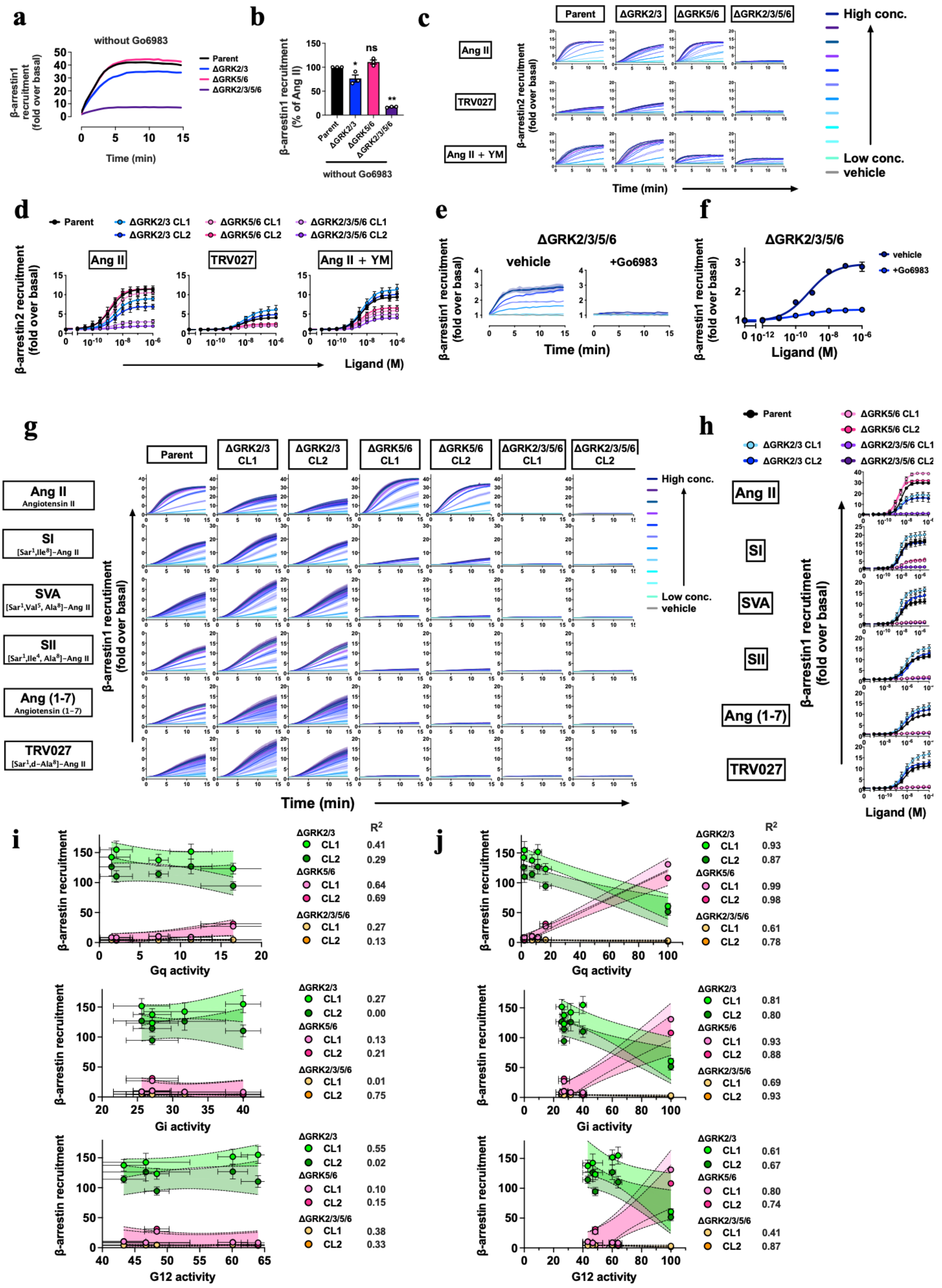
a
b
c
d
e
f
g
h
i
j
without Go6983
Parent
ΔGRK2/3
ΔGRK5/6
ΔGRK2/3/5/6
β-arrestin1 recruitment (fold over basal)
Time (min)
β-arrestin1 recruitment (% of Ang II)
ns
Ang II
TRV027
Ang II + YM
β-arrestin2 recruitment (fold over basal)
High conc.
Low conc.
vehicle
ΔGRK2/3 CL1
ΔGRK2/3 CL2
ΔGRK5/6 CL1
ΔGRK5/6 CL2
ΔGRK2/3/5/6 CL1
ΔGRK2/3/5/6 CL2
Ligand (M)
+Go6983
SI
[Sar1,Ile8]-Ang II
SVA
[Sar1,Val5, Ala8]-Ang II
SII
[Sar1,Ile4, Ala8]-Ang II
Ang (1-7)
Angiotensin (1-7)
[Sar1,d-Ala8]-Ang II
Angiotensin II
β-arrestin recruitment
Gq activity
Gi activity
G12 activity
R2

Supplementary Fig. 3 AT1R biased ligands and their GRK-subtype dependency.

a, b, Representative luminescent kinetics (a) and concentration-response curves (b) of the NanoBiT-β-arrestin assay. The parent and the GRK-deficient cell lines expressing AT1R-Sm and Lg-β-arrestin1 were stimulated with Ang II in the absence of Go6983. Cell lines used were ΔGRK2/3, CL2; ΔGRK5/6, CL2; ΔGRK2/3/5/6, CL2. **c, d,** Representative luminescent kinetics (c) and concentration-response curves (d) of β-arrestin2 recruitment responses. AT1R-Sm and Lg-β-arrestin2 were expressed in the parent and the GRK-deficient cell lines and stimulated with the Ang II (with or without 1 μM YM-254890 pretreatment) or TRV027. **e, f,** Representative luminescent kinetics (e) and concentration-response curves (f) of β-arrestin1-recruitment responses in the presence or absence of Go6983 (3 μM). Cell lines used were ΔGRK2/3/5/6, CL2. **g, h,** Luminescent kinetics (g) and concentration-response curves (h) of β-arrestin1-recruitment responses in the parent and the GRK-deficient cell lines upon stimulation with Ang II and the indicated analogs. **i, j,** Comparison of the β-arrestin recruitment responses and G-protein-coupling activity. The scattered plots represent data from the five ligands except for Ang II (i) or from all of the six ligands (j). We note that the overall correlation coefficient is screwed in the scattered plots containing the six ligands (Supplementary Fig. 3j) owing to the excessive activity of Ang II in both β-arrestin recruitment and G protein signaling. Therefore, the main figure (Fig. 1j) is based on the scattered plots with the five ligands except for Ang II. In Fig. 3e, g, lines and shaded regions represent mean and SEM, respectively, of 3 (e) or 3-6 (g) independent experiments with each performed in duplicate. In Fig. 3b, bars and error bars represent mean and SEM, respectively, of 3 independent experiments with each performed in duplicate. In Fig. 3d, f, h, i, j, symbols and error bars represent mean and SEM, respectively, of 3 (d, f) or 3-6 (h, i, j) independent experiments with each performed in duplicate. In Fig. 3i, j, shaded regions denote 90% confidence of the linear regression analysis. R^2 values are shown at the right. Note that for many data points, vertical error bars are smaller than the size of the symbols, and thus are not visible. In Fig. 3b, * and ** represent P < 0.05 and 0.01, respectively, with one-way ANOVA followed by the Dunnett's test for multiple comparison analysis with reference to the Parent. ns, not significantly different between the groups. See Supplementary statistics data file for additional statistics and exact P values.

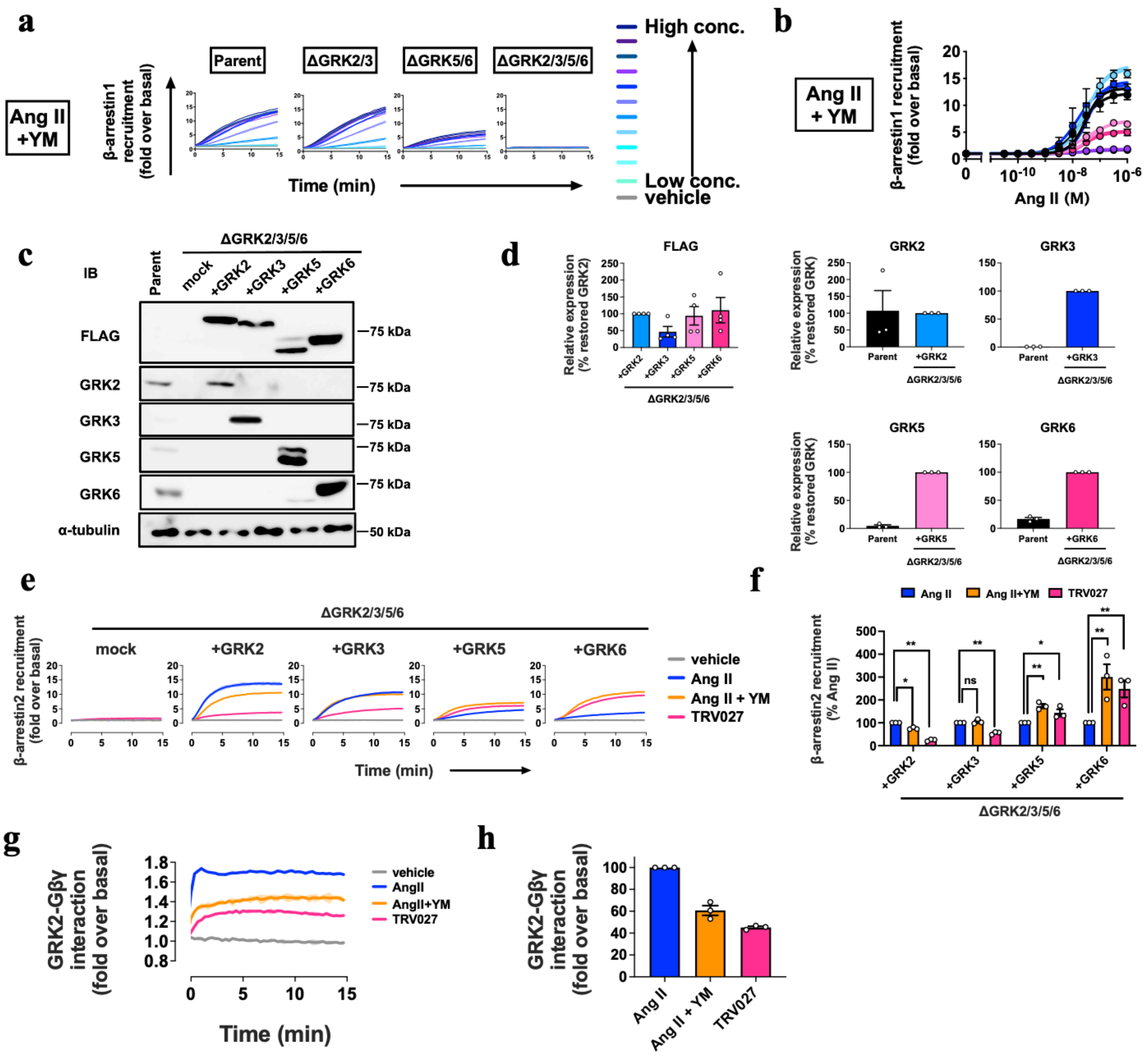

a
Ang II +YM
β-arrestin1 recruitment (fold over basal)
Parent
ΔGRK2/3
ΔGRK5/6
ΔGRK2/3/5/6
Time (min)
High conc.
Low conc.
vehicle
b
Ang II + YM
β-arrestin1 recruitment (fold over basal)
Ang II (M)
c
IB
Parent
ΔGRK2/3/5/6
mock
+GRK2
+GRK3
+GRK5
+GRK6
FLAG
GRK2
GRK3
GRK5
GRK6
α-tubulin
75 kDa
50 kDa
d
FLAG
Relative expression (% restored GRK2)
GRK2
GRK3
GRK5
GRK6
Relative expression (% restored GRK)
e
β-arrestin2 recruitment (fold over basal)
vehicle
Ang II
Ang II + YM
TRV027
Time (min)
f
β-arrestin2 recruitment (% Ang II)
Ang II+YM
g
GRK2-Gβγ interaction (fold over basal)
AngII
AngII+YM
h

Supplementary Fig. 4 Contribution of the individual GRKs to β-arrestin-recruitment responses.

a, b, Representative luminescent kinetics (a) and concentration-response curves (b) of β-arrestin1-recruitment responses in the parent and the GRK-deficient cell lines upon Ang II stimulation pretreated with 1 μM YM-254890. **c, d,** Western blot analyses of re-expressed GRKs. The C-terminally FLAG epitope-tagged GRK constructs were expressed in ΔGRK2/3/5/6 (CL2) cells at a level equivalent to that of endogenous GRK2. Quantification of chemiluminescent band intensity in the western blot analyses (d). Expression levels of the GRK constructs were normalized to that of α-tubulin and shown as relative values to that in the transfected GRK2 (anti-FLAG) or the transfected GRK subtypes (anti-GRK2, 3, 5 and 6). We note that a low MW band was apparent in the GRK5-expressing cells presumably due to degradation of the protein during lysate sample preparation. **e, f,** Assessment of the individual GRK subtypes for their sensitivity to YM-254890. The C-terminally FLAG epitope-tagged GRK constructs was individually expressed along with AT1R-Sm and Lg-β-arrestin2 in ΔGRK2/3/5/6 cells and stimulated with 1 μM Ang II (with or without 1 μM YM-254890 pretreatment) or 1 μM TRV027. **g, h**, AT1R-induced release of Gβγ available to GRK2. AT1R, GRK2-Lg, Sm-Gβ1 and Gγ2 were expressed in the parental cells and stimulated with the indicated conditions. Ang II-induced GRK2-Gβγ interaction signal (h) was set as 100%. Cell lines used were ΔGRK2/3, CL2; ΔGRK5/6, CL2; ΔGRK2/3/5/6, CL2 (a-f). In Fig. 4e, g, lines and shaded regions represent mean and SEM, respectively, of 3 independent experiments with each performed in duplicate. In Fig. 4d, f, h, bars and error bars represent mean and SEM, respectively, of 3 (f, h) or 3-4 (d) independent experiments with each performed in duplicate. In Fig. 4b, symbols and error bars represent mean and SEM, respectively, of 3 (d, f) or 3-6 (h, i, j) independent experiments with each performed in duplicate. In Fig. 4f, * and ** represent $P < 0.05$ and 0.01, respectively, with two-way ANOVA followed by the Dunnett's test for multiple comparison analysis with reference to the Ang II stimulation. ns, not significantly different between the groups. See Supplementary statistics data file for additional statistics and exact P values.

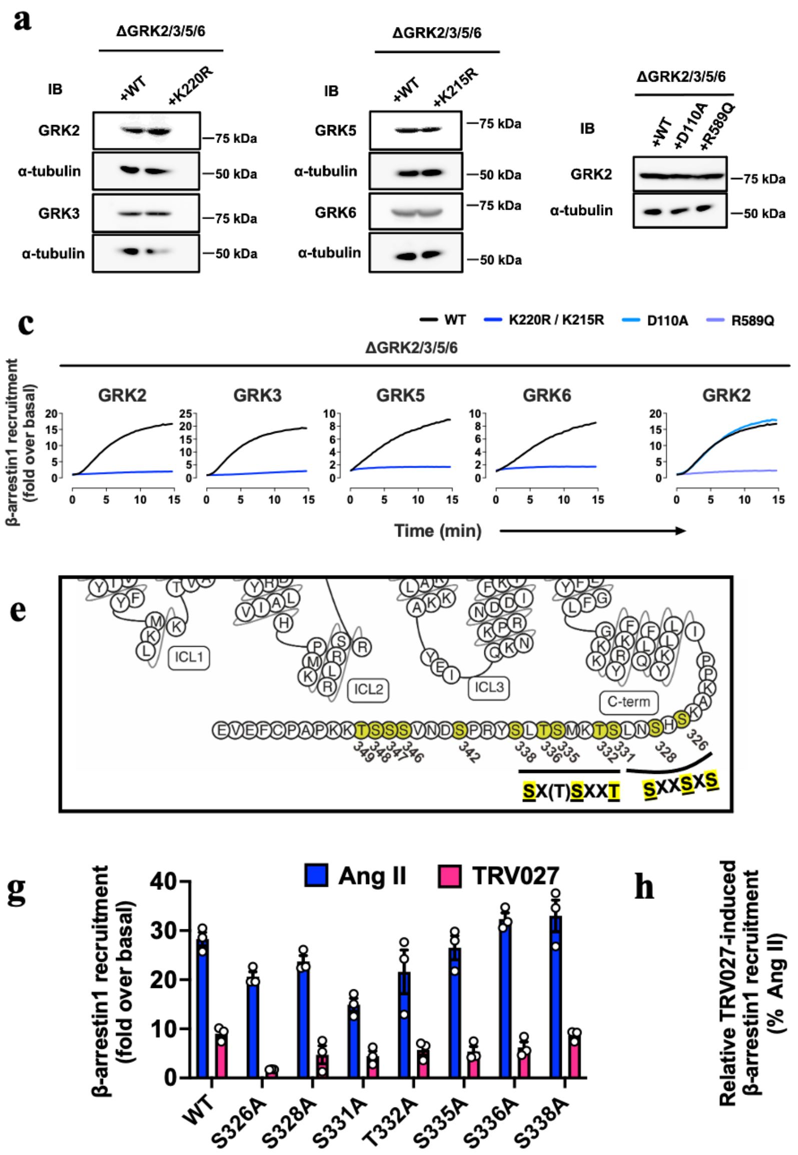

a
ΔGRK2/3/5/6
IB
+WT
+K220R
GRK2
α-tubulin
GRK3
75 kDa
50 kDa
+K215R
GRK5
GRK6
+D110A
+R589Q
b
GRK expression (% WT)
ns
WT
KR
D110A
R589Q
c
WT
K220R / K215R
D110A
R589Q
β-arrestin1 recruitment (fold over basal)
Time (min)
d
β-arrestin1 recruitment (% WT)
**
e
ICL1
ICL2
ICL3
C-term
SX(T)SXXT
SXXSXS
f
Surface expression (% WT MFI)
WT
S326A
S328A
S331A
T332A
S335A
S336A
S338A
g
Ang II
TRV027
h
Relative TRV027-induced β-arrestin1 recruitment (% Ang II)

Supplementary Fig. 5 GRK-dependent AT1R phosphorylation.

a, b, Western blot analyses of re-expressed mutant GRKs. K220R (GRK2 and GRK3) and K215R (GRK5 and GRK6), catalytically impaired mutants; D110A (GRK2), Gαq-binding-deficient mutant; R587Q (GRK2), Gβγ-binding-deficient mutant. **c, d,** Representative luminescent kinetics (c) and concentration-response curves (d) of β-arrestin1-recruitment responses. ΔGRK2/3/5/6 cells transfected with the indicated wild-type or the catalytically impaired GRK construct were subjected to the NanoBiT-β-arrestin recruitment assay. **e,** Ser/Thr residues and the phosphorylation codes in the C-terminal tail of AT1R. Note that there are two potential phosphorylation codes (spanning Ser326-Ser331 and Thr332-Ser338). **f,** Surface expression of WT and the single Ala-substituted AT1R mutants. The parent cells expressing the indicated N-terminally FLAG-tagged AT1R constructs were subjected to the flow cytometry analysis. **g,** β-arrestin1-recruitment responses upon Ang II (1 μM) or 1 μM TRV027 (1 μM) stimulation. **h,** TRV027-induced β-arrestin1 recruitment responses relative to that of Ang II. In Fig. 5b, d, f, g, h, bars and error bars represent mean and SEM, respectively, of 3 independent experiments with each performed in duplicate. In Fig. 5b, d, h, * and ** represent $P < 0.05$ and 0.01, respectively, with one-way (h) or two-way (b, d) ANOVA followed by the Dunnett's (d, h) or Sidak's (b) test for multiple comparison analysis with reference to the WT GRK (b, d) or the WT AT1R (h). ns, not significantly different between the groups. See Supplementary statistics data file for additional statistics and exact *P* values.

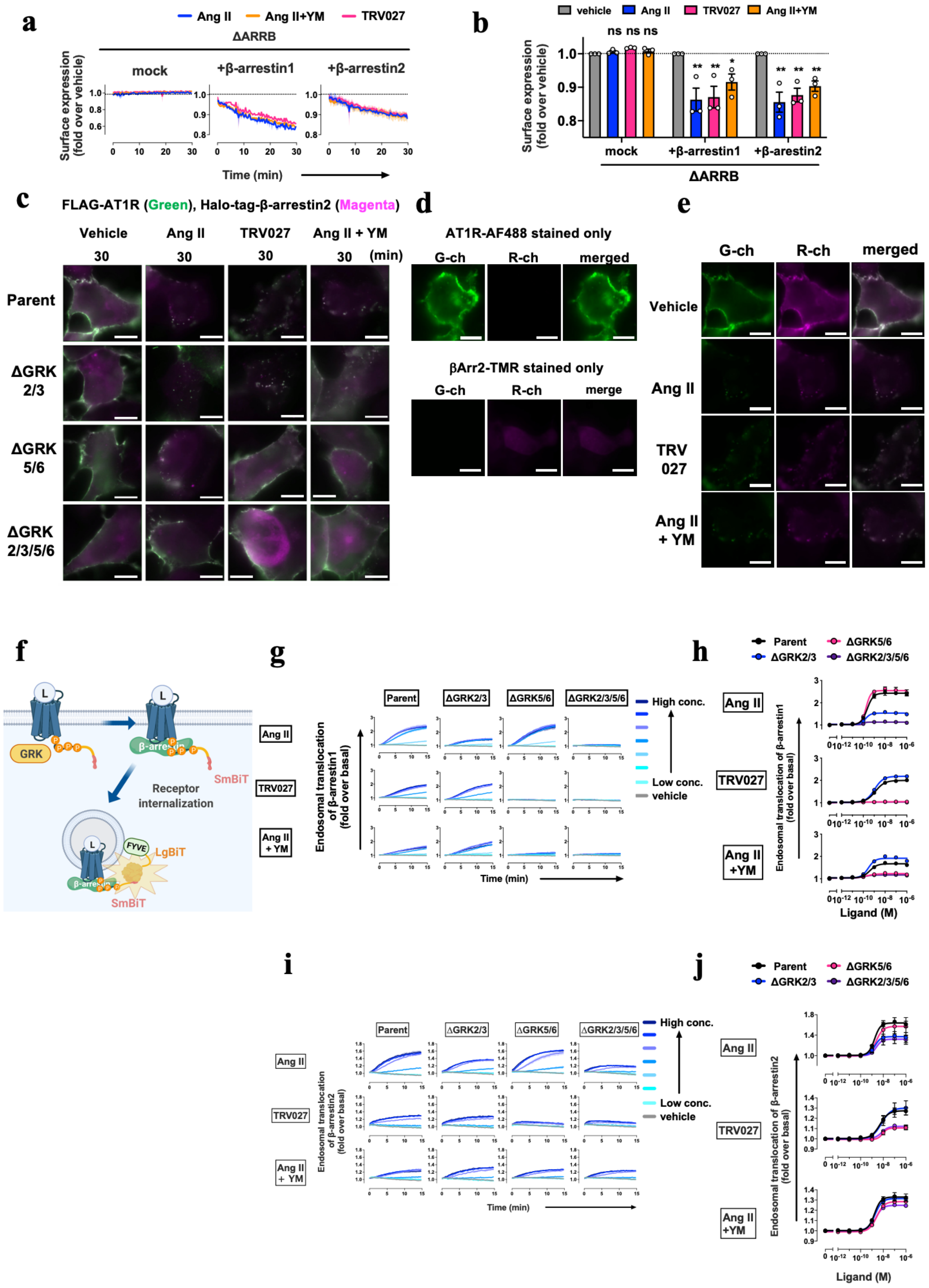

a
Ang II
Ang II+YM
TRV027
ΔARRB
mock
+β-arrestin1
+β-arrestin2
Surface expression (fold over vehicle)
Time (min)
b
vehicle
Ang II
TRV027
Ang II+YM
ns ns ns
mock
+β-arrestin1
+β-arestin2
ΔARRB
c
FLAG-AT1R (Green), Halo-tag-β-arrestin2 (Magenta)
Vehicle
Ang II
TRV027
Ang II + YM
30 (min)
Parent
ΔGRK 2/3
ΔGRK 5/6
ΔGRK 2/3/5/6
d
AT1R-AF488 stained only
G-ch
R-ch
merged
βArr2-TMR stained only
merge
e
Vehicle
TRV 027
f
GRK
β-arrestin
SmBiT
Receptor internalization
FYVE
LgBiT
g
Parent
ΔGRK2/3
ΔGRK5/6
ΔGRK2/3/5/6
High conc.
Low conc.
vehicle
Endosomal translocation of β-arrestin1 (fold over basal)
Time (min)
h
Ligand (M)
i
Endosomal translocation of β-arrestin2 (fold over basal)
j

Supplementary Fig. 6 GRK-dependent AT1R internalization and β-arrestin endosomal translocation.

a, b, Representative luminescent kinetics (a) and quantification (b) of the HiBiT-based AT1R-internalization assay. ΔARRB cells transfected with HiBiT-AT1R along with the empty vector (mock), untagged β-arrestin1 or β-arrestin2 were stimulated with 10 nM Ang II (with or without 1 μM YM-254890) or 100 nM TRV027. At every measurement time point, a ligand-stimulated count was normalized to a vehicle count. **c-e,** Representative images of GRK-dependent AT1R and β-arrestin2 internalization. FLAG-AT1R (green) and SNAP-tag-β-arrestin2 (magenta) were fluorescently labeled (Alexa488 and TMRstar, respectively), treated by the indicated condition followed by fixation, and imaged by the oblique illumination microscopy (c, e). Bleed-through evaluation of the Alexa-Fluor 488 (AF488) and the TMR signals detected in each channel (d). In all panels in (c-e), brightness and contrast of the images were adjusted in the same min and max values. f-j**,** Schematic representation (f), representative luminescent kinetics (g, i) and concentration-response curves (h, j) of the NanoBiT-β-arrestin endosomal translocation assay. A NanoBiT pair consisting of the SmBiT-fused β-arrestin1 (g, h) or β-arrestin2 (i, j) construct and the LgBiT-fused FYVE domain of Endofin, an endosome-localizing protein, was expressed along with the untagged AT1R and AT1R ligand-induced β-arrestin translocation was monitored. Cell lines used were ΔGRK2/3, CL2; ΔGRK5/6, CL2; ΔGRK2/3/5/6, CL2 (g-j). In Fig. 6g, i, lines and shaded regions represent mean and SEM, respectively, of 3 independent experiments with each performed in duplicate. In Fig. 6b, bars and error bars represent mean and SEM, respectively, of 3 independent experiments with each performed in duplicate. In Fig. 6c-e, representative images of 20-32 cells were shown. In Fig. 6h, j, symbols and error bars represent mean and SEM, respectively, of 3 independent experiments with each performed in duplicate. In Fig. 6b, * and ** represent P < 0.05 and 0.01, respectively, with one-way ANOVA followed by the Dunnett's test for multiple comparison analysis with reference to the vehicle stimulation. ns, not significantly different between the groups. See Supplementary statistics data file for additional statistics and exact P values.

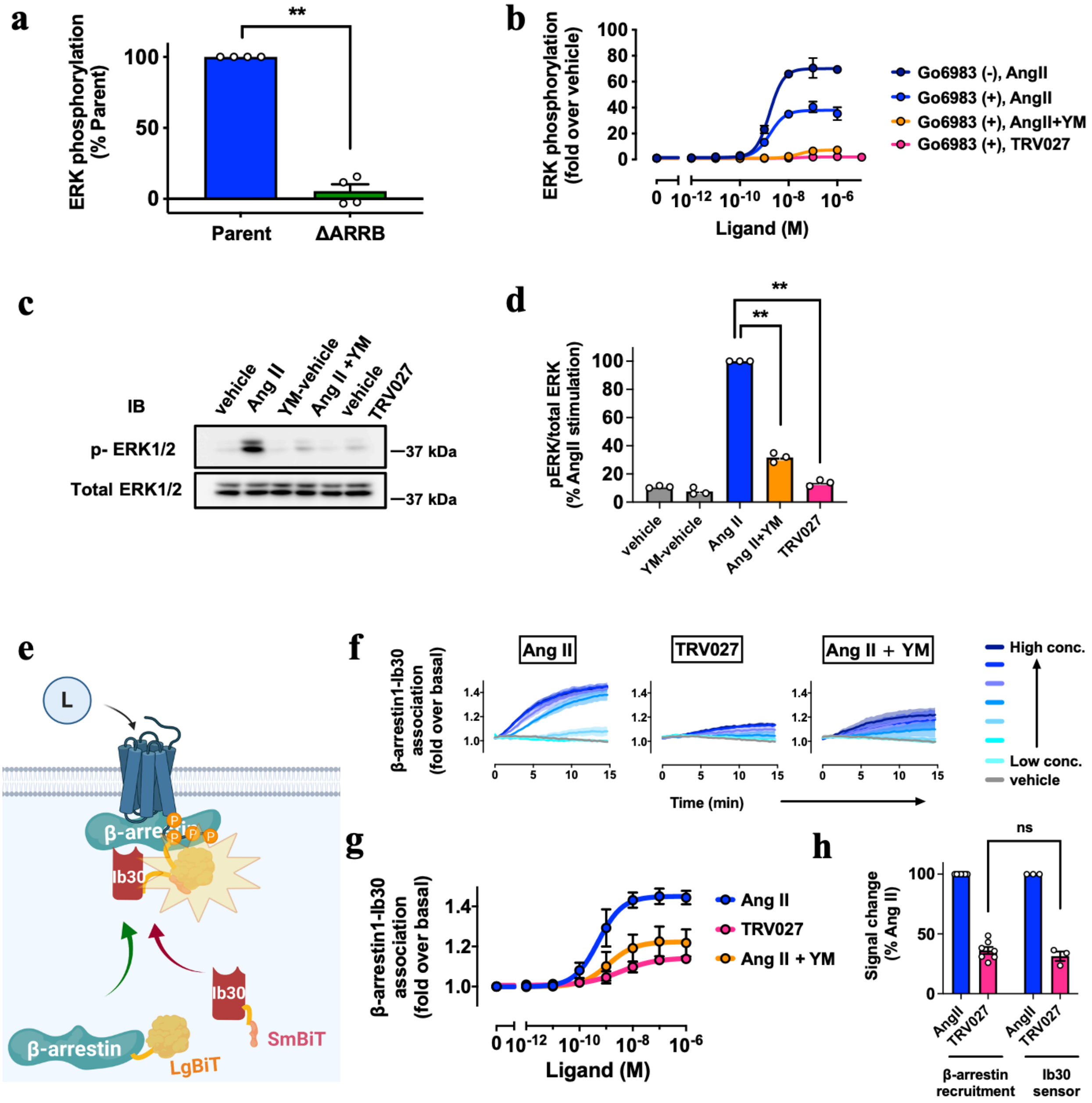
a
ERK phosphorylation (% Parent)
**
Parent
ΔARRB
b
ERK phosphorylation (fold over vehicle)
Ligand (M)
Go6983 (-), AngII
Go6983 (+), AngII
Go6983 (+), AngII+YM
Go6983 (+), TRV027
c
IB
vehicle
Ang II
YM-vehicle
Ang II +YM
vehicle
TRV027
p- ERK1/2
Total ERK1/2
37 kDa
37 kDa
d
pERK/total ERK (% AngII stimulation)
vehicle
YM-vehicle
Ang II
Ang II+YM
TRV027
e
L
β-arrestin
Ib30
Ib30
β-arrestin
LgBiT
SmBiT
f
β-arrestin1-Ib30 association (fold over basal)
Ang II
TRV027
Ang II + YM
Time (min)
High conc.
Low conc.
vehicle
g
β-arrestin1-Ib30 association (fold over basal)
Ligand (M)
Ang II
TRV027
Ang II + YM
h
Signal change (% Ang II)
ns
AngII
TRV027
AngII
TRV027
β-arrestin recruitment
Ib30 sensor

Supplementary Fig. 7 ERK phosphorylation and β-arrestin conformational change under the Gq-inactivated condition.

a, Validation of β-arrestin-mediated pERK responses. The parent or the β-arrestin1/2-deficient (ΔARRB) cells expressing AT1R were pretreated with 3 μM Go6983 and stimulated with 1 μM Ang II for 30 min. Phosphorylated ERK levels were quantified by the AlphaLISA-based assay and shown as normalized signals. **b,** Concentration-response curves for pERK responses. The parent cells expressing AT1R were pretreated with or without 3 μM Go6983 and/or 1 μM YM-254890 and stimulated with the titrated concentrations of Ang II or TRV027 for 30 min. **c, d,** Western blot-based ERK phosphorylation analysis. Representative blot images of phosphorylated ERK1/2 and total ERK1/2 by the indicated treatment (c). Quantification of chemiluminescent band intensities (d). For each experiment, pERK responses were normalized to that of Ang II. **e-g**, Schematic representation (e), kinetics (f) and concentration-response curves (g) of the NanoBiT-β-arrestin-conformation sensor. AT1R ligand-induced β-arrestin1 conformational change was monitored by the single-chain Ib30 sensor. The parental cells expressing the Lg-β-arrestin1 and Sm-Ib30 along with AT1R were stimulated with the titrated concentrations of Ang II (with or without 1 μM YM-254890 pretreatment) or TRV027. **h,** Comparison of β-arrestin1 responses between recruitment and Ib30-recognizable conformational change. For each assay, TRV027-induced responses were normalized to that of Ang II. In Fig. 7f, lines and shaded regions represent mean and SEM, respectively, of 3 independent experiments with each performed in duplicate. In Fig. 7a, d, h, bars and error bars represent mean and SEM, respectively, of 3 (d) or 4 (a) or 3-7 (h) independent experiments with each performed in duplicate. In Fig. 7b, g, symbols and error bars represent mean and SEM, respectively, of 3 independent experiments with each performed in duplicate. In Fig. 7a, d, h, * and ** represent $P < 0.05$ and 0.01, respectively, with two-tailed multiple t-test (a) or one-way (d) or Two-way (h) ANOVA followed by the Dunnett's test for multiple comparison analysis with reference to the Ang II stimulation. ns, not significantly different between the groups. See Supplementary statistics data file for additional statistics and exact *P* values.

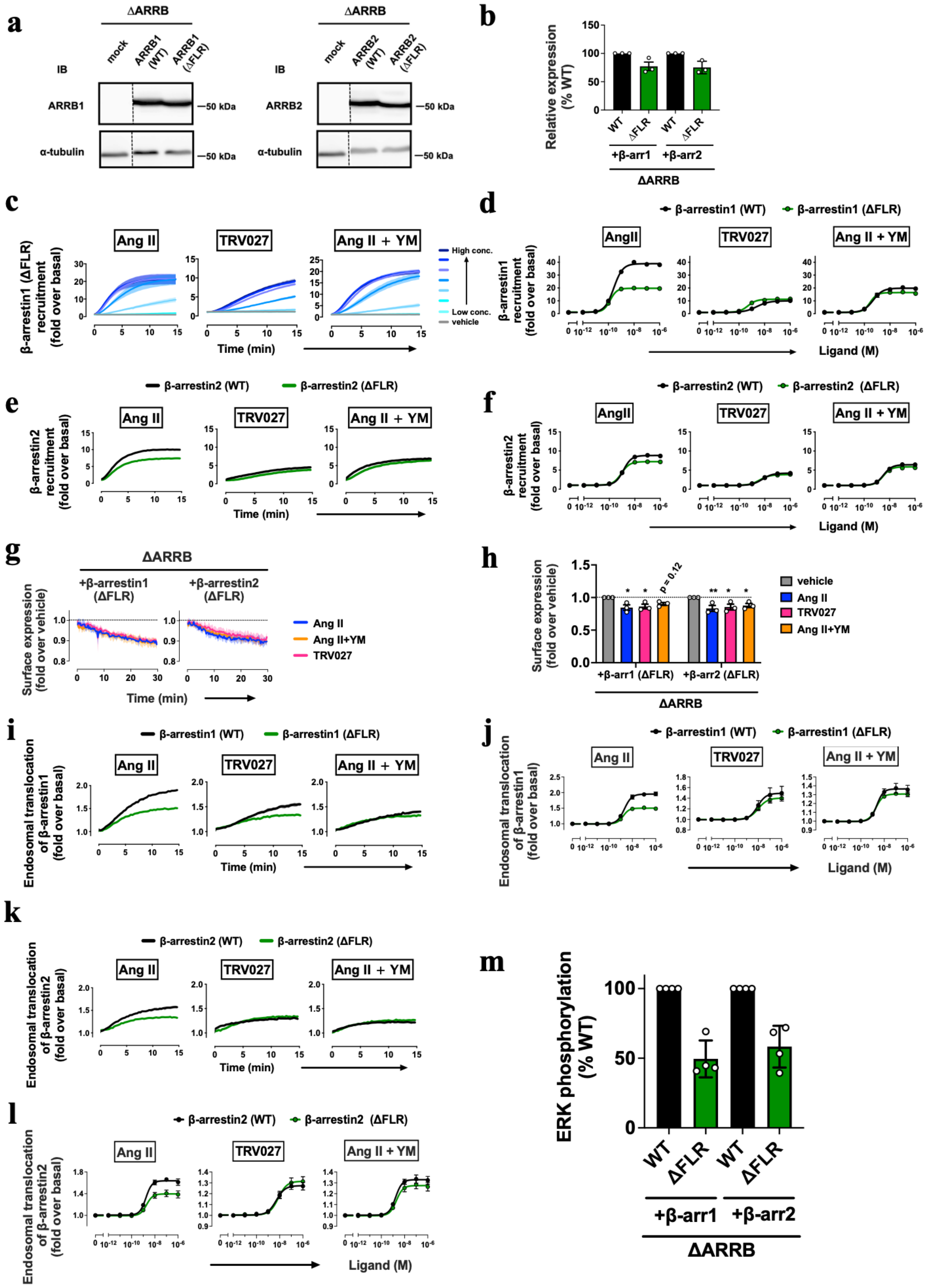
a
ΔARRB
mock
ARRB1 (WT)
ARRB1 (ΔFLR)
IB
ARRB1
α-tubulin
50 kDa
ARRB2
ARRB2 (WT)
ARRB2 (ΔFLR)
b
Relative expression (% WT)
WT
ΔFLR
+β-arr1
+β-arr2
c
β-arrestin1 (ΔFLR) recruitment (fold over basal)
Ang II
TRV027
Ang II + YM
High conc.
Low conc.
vehicle
Time (min)
d
β-arrestin1 (WT)
β-arrestin1 (ΔFLR)
AngII
Ligand (M)
e
β-arrestin2 (WT)
β-arrestin2 (ΔFLR)
β-arrestin2 recruitment (fold over basal)
f
g
+β-arrestin1 (ΔFLR)
+β-arrestin2 (ΔFLR)
Surface expression (fold over vehicle)
Ang II
Ang II+YM
TRV027
h
vehicle
p = 0.12
+β-arr1 (ΔFLR)
+β-arr2 (ΔFLR)
i
Endosomal translocation of β-arrestin1 (fold over basal)
j
k
Endosomal translocation of β-arrestin2 (fold over basal)
l
m
ERK phosphorylation (% WT)

Supplementary Fig. 8 FLR-dependent β-arrestin functions.

a, b, Western blot analyses of FLR-truncated (ΔFLR) β-arrestin expression. The N-terminally SmBiT-fused β-arrestin constructs were expressed in ΔARRB cells. Expression levels of the re-expressed β-arrestin were normalized to that of α-tubulin and shown as relative values to that in WT β-arrestin. **c-f,** Luminescent kinetics (c), representative luminescent kinetics (e) and concentration-response curves (d, f) of the NanoBiT-β-arrestin-recruitment assay for ΔFLR β-arrestin mutants. The parent cells expressing AT1R-Sm along with the indicated Lg-β-arrestin constructs (WT or ΔFLR; β-arrestin1 or β-arrestin2) were subjected to the NanoBiT assay using Ang II and TRV027. Shaded regions denote SEM (c; $n = 3$). Responses of the WT β-arrestin construct that were examined in parallel with the ΔFLR construct were overlaid (d-f). **g, h,** Representative luminescent kinetics (g) and quantification (h) of the HiBiT-based AT1R-internalization assay. ΔARRB cells expressing AT1R along with ΔFLR β-arrestin1 or ΔFLR β-arrestin2 were stimulated with 10 nM Ang II (with or without 1 μM YM-254890 pretreatment) or 100 nM TRV027. At every measurement time point, a ligand-stimulated count was normalized to a vehicle count (g). **i-l,** Representative luminescent kinetics (i, k) and concentration-response curves (j, l) of the NanoBiT-β-arrestin endosomal translocation assay. The parent cells expressing AT1R and Endo-Lg along the indicated Sm-β-arrestin constructs (WT or ΔFLR; β-arrestin1 or β-arrestin2) were subjected to the NanoBiT assay using Ang II and TRV027. **m,** The AlphaLISA-based ERK phosphorylation assay. ΔARRB cells expressing AT1R along with Sm-β-arrestin1 or Sm-β-arrestin2 construct were stimulated with 100 nM Ang II for 30 min in the presence of 3 μM Go6983. For each experiment, pERK signals in ΔFLR β-arrestin were normalized to that of WT β-arrestin. In Fig. 8c, lines and shaded regions represent mean and SEM, respectively, of 3 independent experiments with each performed in duplicate. In Fig. 8b, h, m, bars and error bars represent mean and SEM, respectively, of 3 (b, h) or 4 (m) independent experiments with each performed in duplicate. In Fig. 8d, f, j, l, symbols and error bars represent mean and SEM, respectively, of 3 independent experiments with each performed in duplicate. In Fig. 8h, m, * and ** represent $P < 0.05$ and 0.01, respectively, with two-way ANOVA followed by the Dunnett's test for multiple comparison analysis with reference to the vehicle stimulation (h) or WT β-arrestin (m). ns, not significantly different between the groups. See Supplementary statistics data file for additional statistics and exact *P* values.

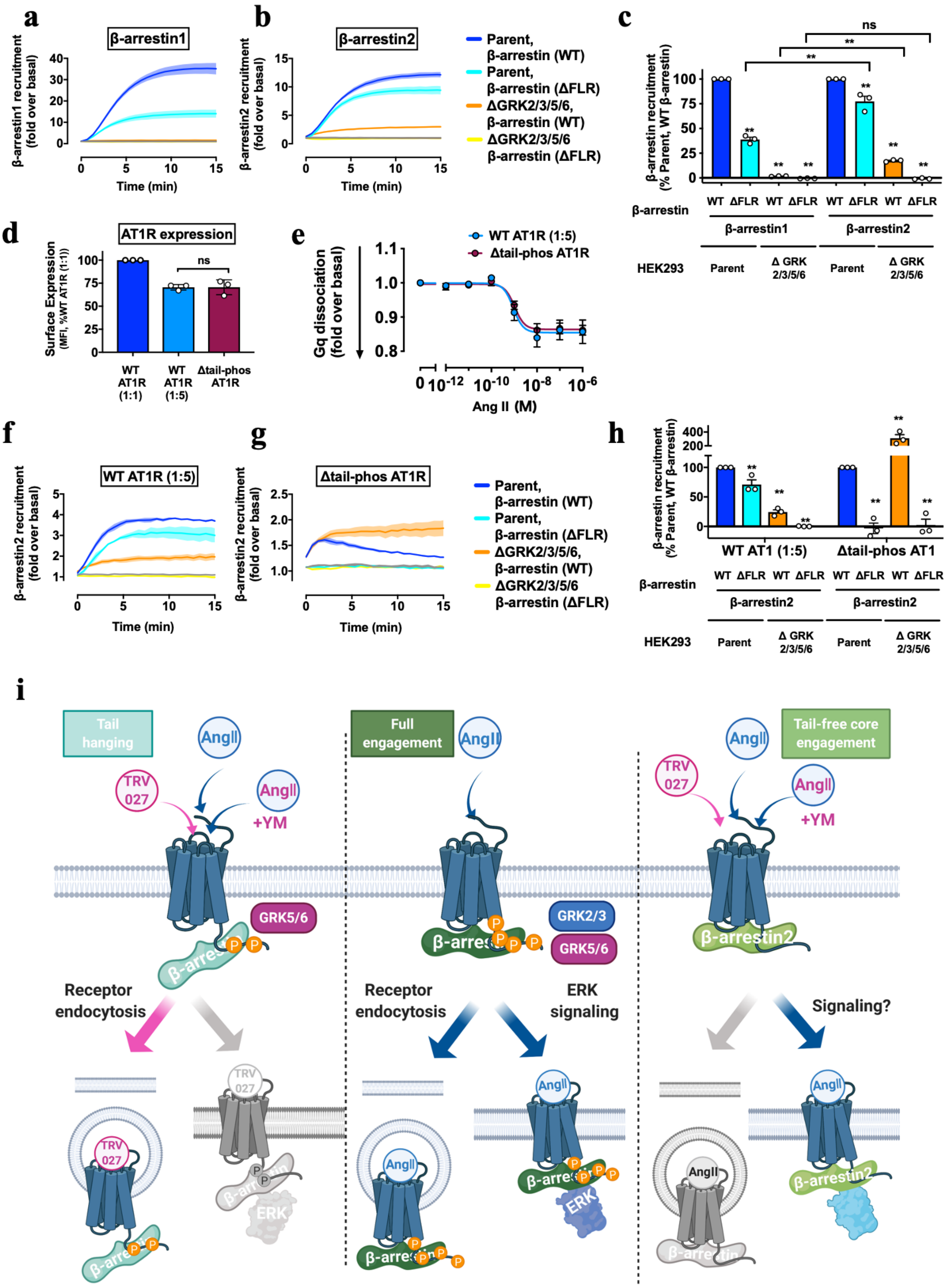
a
β-arrestin1
β-arrestin1 recruitment (fold over basal)
Time (min)
b
β-arrestin2
β-arrestin2 recruitment (fold over basal)
Time (min)
Parent, β-arrestin (WT)
Parent, β-arrestin (ΔFLR)
ΔGRK2/3/5/6, β-arrestin (WT)
ΔGRK2/3/5/6 β-arrestin (ΔFLR)
c
β-arrestin recruitment (% Parent, WT β-arrestin)
ns
**
β-arrestin
WT ΔFLR WT ΔFLR WT ΔFLR WT ΔFLR
β-arrestin1
β-arrestin2
HEK293
Parent
Δ GRK 2/3/5/6
d
AT1R expression
Surface Expression (MFI, %WT AT1R (1:1))
ns
WT AT1R (1:1)
WT AT1R (1:5)
Δtail-phos AT1R
e
Gq dissociation (fold over basal)
WT AT1R (1:5)
Δtail-phos AT1R
Ang II (M)
f
WT AT1R (1:5)
β-arrestin2 recruitment (fold over basal)
Time (min)
g
Δtail-phos AT1R
β-arrestin2 recruitment (fold over basal)
Time (min)
Parent, β-arrestin (WT)
Parent, β-arrestin (ΔFLR)
ΔGRK2/3/5/6, β-arrestin (WT)
ΔGRK2/3/5/6 β-arrestin (ΔFLR)
h
β-arrestin recruitment (% Parent, WT β-arrestin)
WT AT1 (1:5)
Δtail-phos AT1
β-arrestin
WT ΔFLR WT ΔFLR WT ΔFLR WT ΔFLR
β-arrestin2
β-arrestin2
HEK293
Parent
Δ GRK 2/3/5/6
i
Tail hanging
AngII
TRV 027
AngII +YM
GRK5/6
Receptor endocytosis
Full engagement
GRK2/3
GRK5/6
Receptor endocytosis
ERK signaling
Tail-free core engagement
β-arrestin2
Signaling?
ERK

Supplementary Fig. 9 AT1R phosphorylation-independent, tail-free core engagement of β-arrestin.

a-c, Luminescent kinetics (a, b) and quantification (c) of the NanoBiT-β-arrestin recruitment assay. The parent or ΔGRK2/3/5/6 (CL2) cells expressing AT1R-Sm along with the indicated Lg-β-arrestin constructs (WT or ΔFLR; β-arrestin1 or β-arrestin2) were stimulated with 1 μM Ang II. **d,** Cell surface expression of Δtail-phos AT1R assessed by the flow cytometry analysis. Δtail-phos refers to the AT1R mutant with substitution of all of the 12 Ser/Thr residues (Supplementary Fig. 5e). Note that 1:5 refers to a diluted plasmid volume (i.e., 20%) of the default condition (see Methods) and the expression level of Δtail-phos condition was equivalent to that of WT (1:5). **e,** Gq activation of Δtail-phos AT1R assessed by the NanoBiT-G-protein dissociation assay. The NanoBiT-Gq protein consisting of the Gαq-Lg, the untagged Gβ1 and the Sm-Gγ2 (C68S) subunits dissociates upon GPCR ligand stimulation, thereby causing decrease in luminescent signal. **f-h,** Representative luminescent kinetics (f, g) and quantification (h) of the NanoBiT-β-arrestin recruitment assay. **i,** Schematic illustration of the three types of β-arrestin engagement (tail-hanging, full engagement and tail-free core engagement) and their distinct functions. In Fig. 9a, b, f, g, lines and shaded regions represent mean and SEM, respectively, of 3 independent experiments with each performed in duplicate. In Fig. 9c, d, h, bars and error bars represent mean and SEM, respectively, of 3 independent experiments with each performed in duplicate. In Fig. 9e, symbols and error bars represent mean and SEM, respectively, of 3 independent experiments with each performed in duplicate. In Fig. 9c, d, h, * and ** represent $P < 0.05$ and 0.01, respectively, with two-tailed t-test (c) or with two-way ANOVA followed by the Dunnett's test (c, h) for multiple comparison analysis ($n = 3$ in dots; asterisks on the top of bars, with reference to WT in Parent; asterisks on the horizontal lines, between the indicated groups). ns, not significantly different between the groups. See Supplementary statistics data file for additional statistics and exact *P* values.

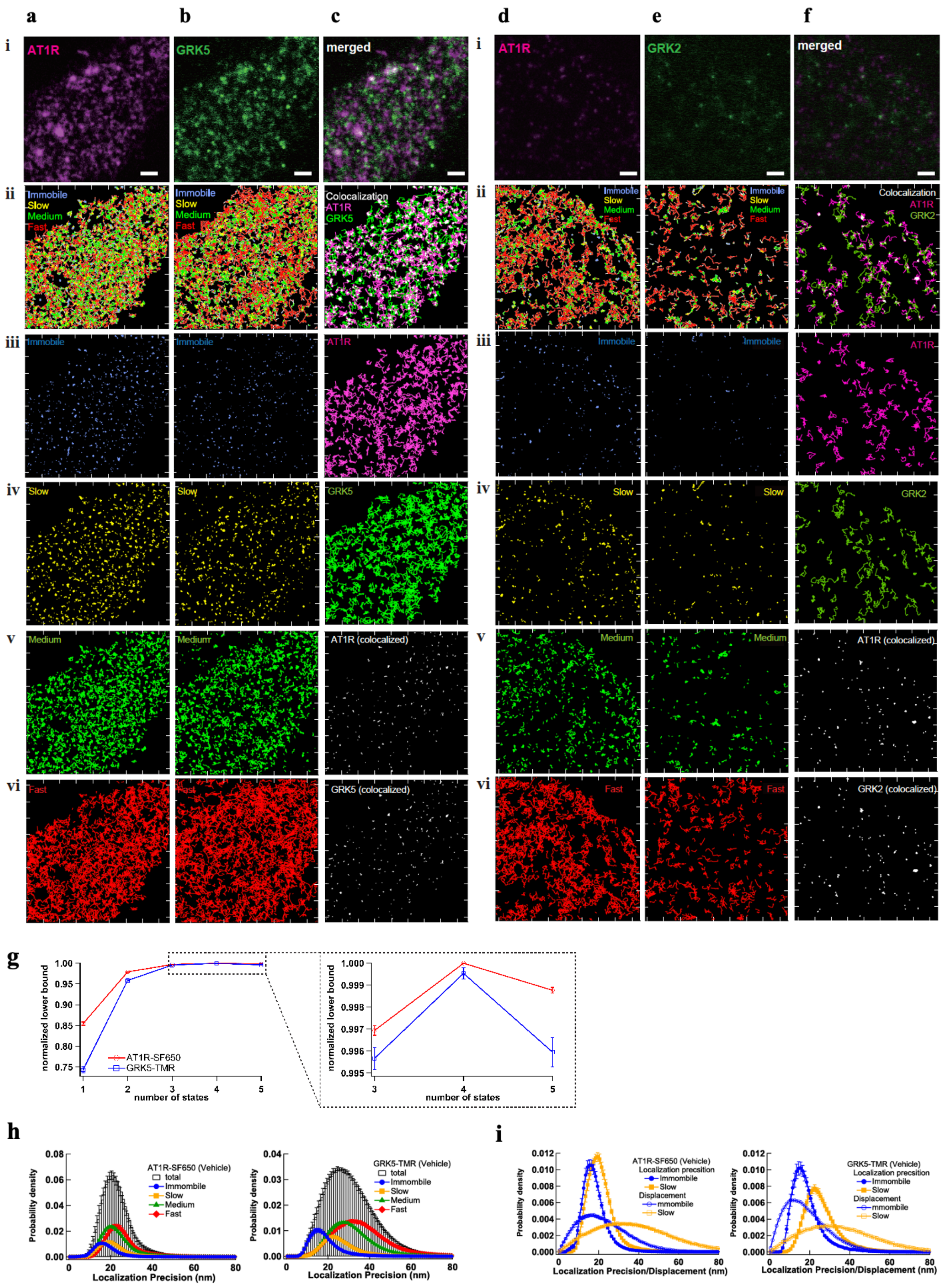

a
b
c
d
e
f
AT1R
GRK5
merged
AT1R
GRK2
merged
Immobile
Slow
Medium
Fast
Colocalization
AT1R
GRK5
GRK2
AT1R (colocalized)
GRK5 (colocalized)
GRK2 (colocalized)
g
normalized lower bound
number of states
AT1R-SF650
GRK5-TMR
h
Probability density
Localization Precision (nm)
AT1R-SF650 (Vehicle)
GRK5-TMR (Vehicle)
total
i
Localization Precision/Displacement (nm)
Localization precsition
Displacement

Supplementary Fig. 10. Dual-color SMT analysis of AT1R-GRK5 and AT1R-GRK2.

a-f, TIRF microscopic image of ΔGRK2/3/5/6 (CL2) cell expressing SF650-labeled AT1R (magenta) and TMR-labeled GRK5 (green, a-c) or TMR-labeled GRK2 (green, d-f). AT1R (a, d), GRK5 (b) and GRK2 (e) molecules and their divided diffusion states are shown in separate images: the original TIRF image (i) and all (ii), the immobile state (iii), the slow state (iv), the medium state (v), and the fast state (vi) trajectories.AT1R and GRK images were merged and colocalization is shown in white (c, f). (ii-vi) Trajectories with at least one colocalized frame were extracted from (a, magenta) and (b, green). The colocalized frame is shown in white dots with a line. (ii) all, (iii) AT1R, (iv) GRK, (v) AT1R-colocalized frame, (vi) GRK-colocalized frame. (g) Numbers of HMM states and its selection. Single-molecule-tracking data for AT1R and GRK5 molecules (labeled with SF650 and TMR fluorophores, respectively) were classified based on the one- to five-state HMMs. Note that the four-state model, although the differences among the three to five states were modest, gave the top score for the lower bound values. **h,** ΔGRK2/3/5/6 (CL2) cells expressing SF650-labeled AT1R and TMR-labeled GRK5 in the absence of ligand stimulation (vehicle) were monitored for their single-molecule behaviors. The observed molecules were divided into four states (immobile, slow, medium and fast) according to their trajectories. Thereafter, localization precisions for each state and for total particles were calculated and plotted as histograms. **i,** Same as in (h), but localization precisions and displacements for the immobile and the slow states were calculated and plotted as histograms. In Fig. 10a-f, representative images of 20-29 cells were shown. In Fig. 10g, h, i, symbols and error bars represent mean and SEM, respectively, of 20-29 cells.

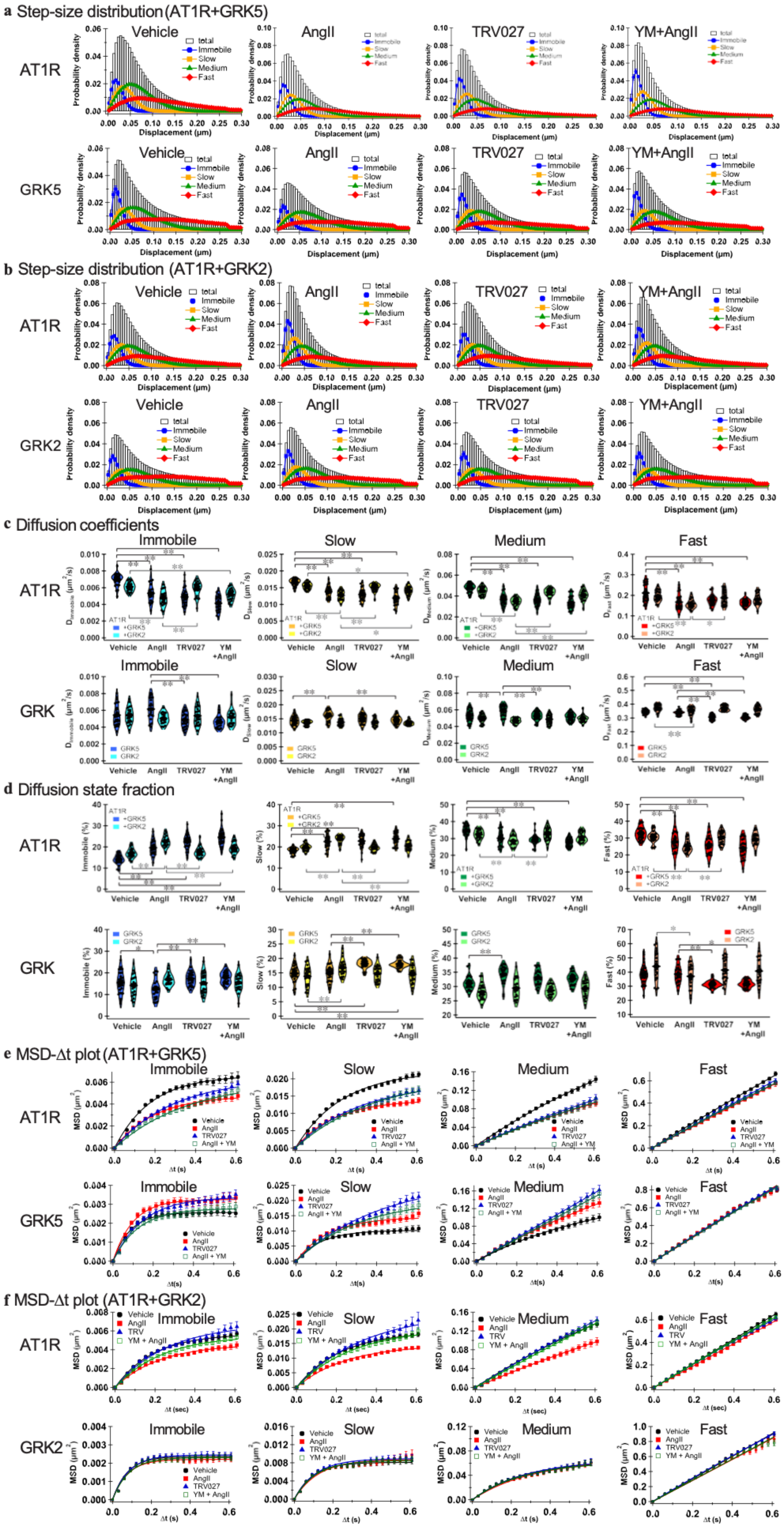
a Step-size distribution (AT1R+GRK5)
AT1R
GRK5
Vehicle
AngII
TRV027
YM+AngII
total
Immobile
Slow
Medium
Fast
Probability density
Displacement (μm)
b Step-size distribution (AT1R+GRK2)
AT1R
GRK2
c Diffusion coefficients
Immobile
Slow
Medium
Fast
AT1R
GRK
+GRK5
+GRK2
Vehicle
AngII
TRV027
YM +AngII
d Diffusion state fraction
Immobile (%)
Slow (%)
Medium (%)
Fast (%)
e MSD-Δt plot (AT1R+GRK5)
MSD (μm²)
Δt (s)
AngII + YM
f MSD-Δt plot (AT1R+GRK2)
TRV
YM + AngII
Δt (sec)

Supplementary Fig. 11 Single-molecule diffusion behaviors of AT1R and GRK5 molecules.

SF650-labeled AT1R and TMR-labeled GRK5 (a, c, d, e) or GRK2 (b, c, d, f) were expressed in ΔGRK2/3/5/6 (CL2) cells. **a, b,** Displacement histogram of the trajectories of AT1R and GRK5 (a) and of AT1R and GRK2 (b) divided into the four states under the indicated condition (Vehicle, 1 μM Ang II, 1 μM TRV027 or 1 μM Ang II pretreated with 1 μM YM-254890). **c, d,** Comparison of diffusion coefficient (c) and fraction (d) of AT1R and GRK2 or GRK5 molecules under the indicated stimulations in each diffusion state. **e, f,** MSD-Δt plots of AT1R and GRK5 (e), and of AT1R and GRK2 (f). MSD-Δt plots of the trajectories of AT1R and GRK molecules under the indicated stimulations. In Fig. 11a, b, e, f, symbols and error bars represent mean and SEM, respectively, of 20-29 cells. In Fig. 11c, d, shaded regions represent a histogram of 20-29 cells. * and ** represent $P < 0.05$ and 0.01, respectively, with one-way ANOVA followed by the Tukey HSD test among 4 groups (n = 20-29 cells). ns, not significantly different between the groups. See Supplementary statistics data file for additional statistics and exact P values.

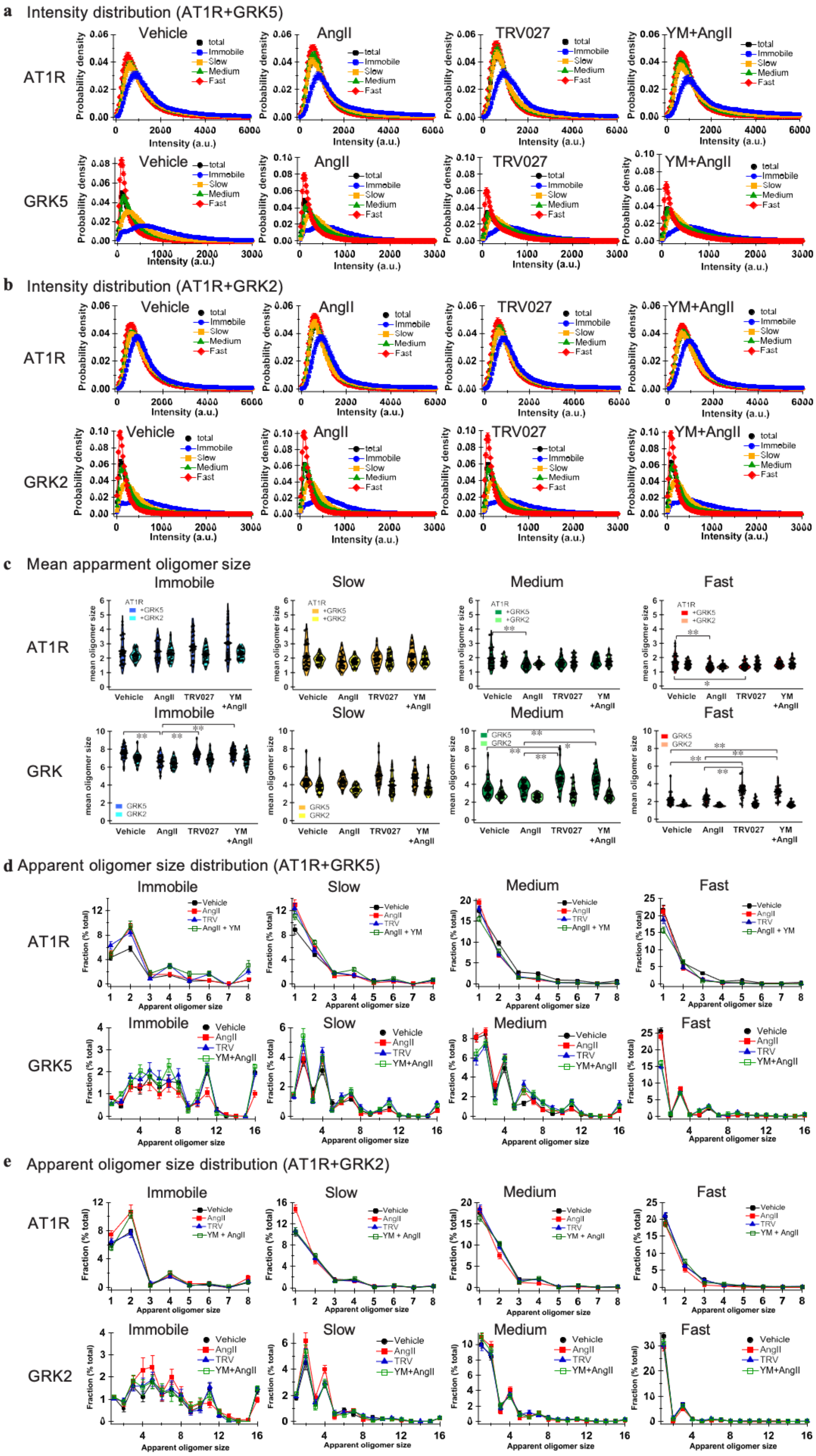

a Intensity distribution (AT1R+GRK5)
AT1R
GRK5
Vehicle
AngII
TRV027
YM+AngII
total
Immobile
Slow
Medium
Fast
Probability density
Intensity (a.u.)
b Intensity distribution (AT1R+GRK2)
GRK2
c Mean apparment oligomer size
mean oligomer size
+GRK5
+GRK2
GRK
YM +AngII
d Apparent oligomer size distribution (AT1R+GRK5)
Fraction (% total)
Apparent oligomer size
TRV
AngII + YM
e Apparent oligomer size distribution (AT1R+GRK2)
YM + AngII

Supplementary Fig. 12 Apparent oligomer size of AT1R and GRK5 molecules.

SF650-labeled AT1R and TMR-labeled GRK5 (a, c, d) or GRK2 (b, c, e) were expressed in ΔGRK2/3/5/6 (CL2) cells. **a, b,** Histogram of fluorescence intensity distribution of SF650-labeled AT1R (upper panels) and of TMR-labeled GRK2 or GRK5 (lower panels) divided into the four diffusion states under the indicated condition (Vehicle, 1 μM Ang II, 1 μM TRV027 or 1 μM Ang II pretreated with 1 μM YM-254890). **c,** Mean apparent oligomer size of AT1R (upper panels) and of GRKs (lower panels) in each diffusion state. **d, e,** Distribution of the apparent oligomer size of AT1R (upper panels) and of GRKs (lower panels). For each diffusion state, distribution was estimated from the intensity histogram based on the sum of the Gaussian functions. The sum of the fractions of all diffusion states were represented as 100%. In Fig. 12a, b, e, f, symbols and error bars represent mean and SEM, respectively, of 20-29 cells. In Fig. 12c, d, shaded regions represent a histogram of 20-29 cells. * and ** represent $P < 0.05$ and 0.01, respectively, with one-way ANOVA followed by the Tukey HSD test among 4 groups (n = 20-29 cells). ns, not significantly different between the groups. See Supplementary statistics data file for additional statistics and exact P values.

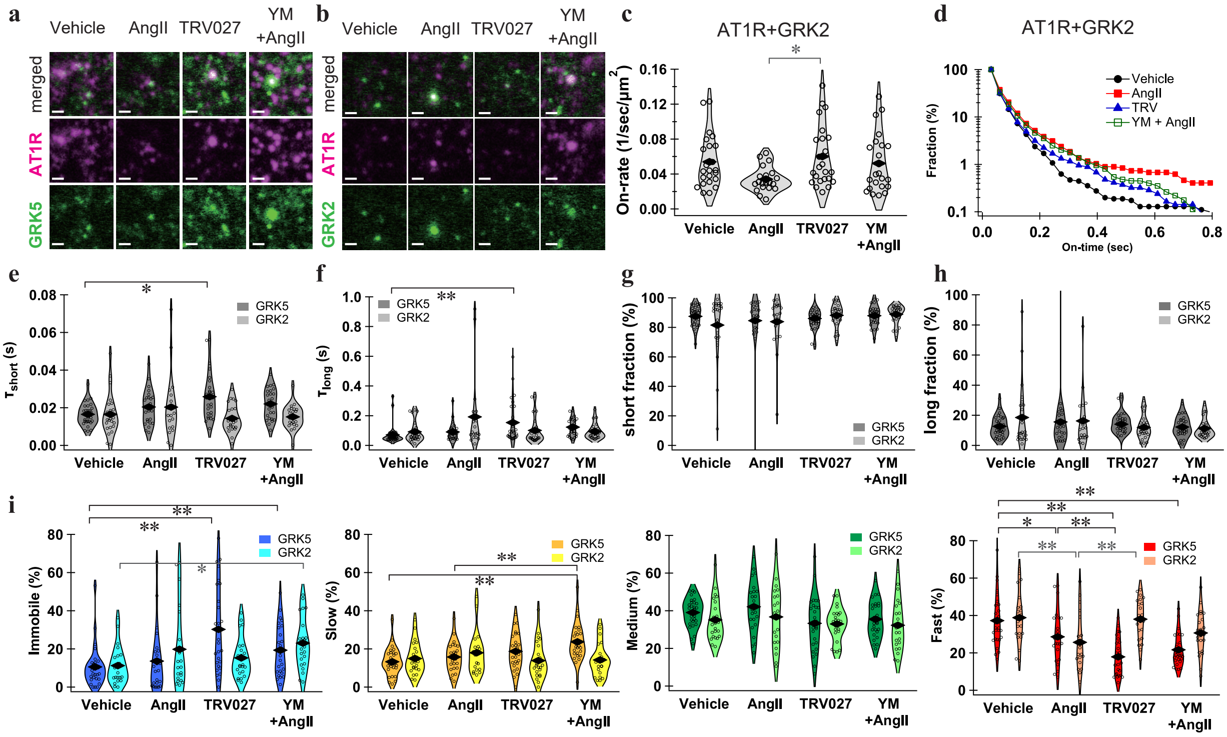

Supplementary Fig. 13 Single-molecule behaviors of colocalized AT1R and GRK molecules.

a, b, Representative image of AT1R-GRK5 (a) and of AT1R-GRK2 (b) colocalization. Scale bar: 1 µm. **c, d,** AT1R-GRK2 association (c, on-event) and dissociation (d, colocalization duration) rate were estimated from all trajectories. **e-h,** Time constants (e, τ_{short}; f, τ_{long}) and their fractions (g, short; h, long) of AT1R/GRK colocalization were estimated from curve fitting based on the double-exponential function. **i,** Fractions of the diffusion states of AT1R molecules colocalized with GRK5 or GKR2 molecules by the indicated condition. In Fig. 13a, b, representative images of 20-29 cells were shown. In Fig. 13d, Symbols represent mean of all trajectories in 20-29 cells. In Fig. 13c, e-j, shaded regions represent a histogram of 20-29 cells. * and ** represent $P < 0.05$ and 0.01, respectively, in the one-way ANOVA with followed by the Tukey HSD test among 4 groups (n = 20-29 cells). ns, not significantly different between the groups. See Supplementary statistics data file for additional statistics and exact P values.

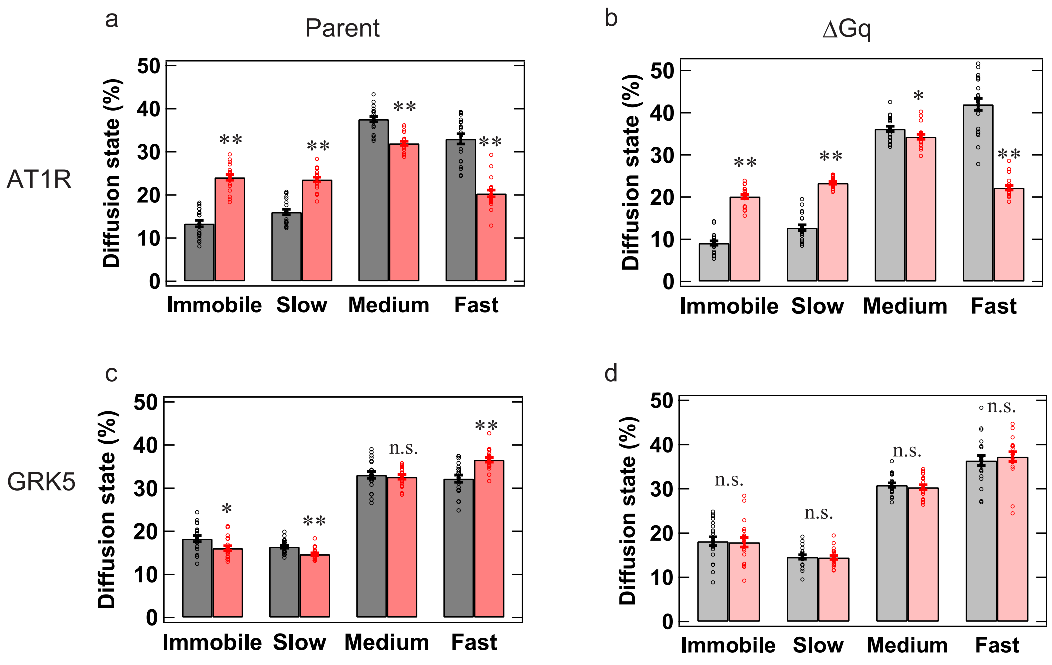

Supplementary Fig. 14 Diffusion state fractions of AT1R and GRK5 molecules in ΔGq cells

The fractions of the diffusion states of AT1R (a, b) and GRK5 (c, d) in parent cells (a, c) or in ΔGq cells. In each panel, comparison was performed between vehicle and Ang II (1 µM) stimulation. Bars and error bars represent mean and SEM, respectively, from TIRF microscopy data in 20 cells. * and ** represent $P < 0.05$ and 0.01, respectively, with the Welch's t-test (two-tailed, $n = 20$ cells). See Supplementary statistics data file for additional statistics and exact P values.

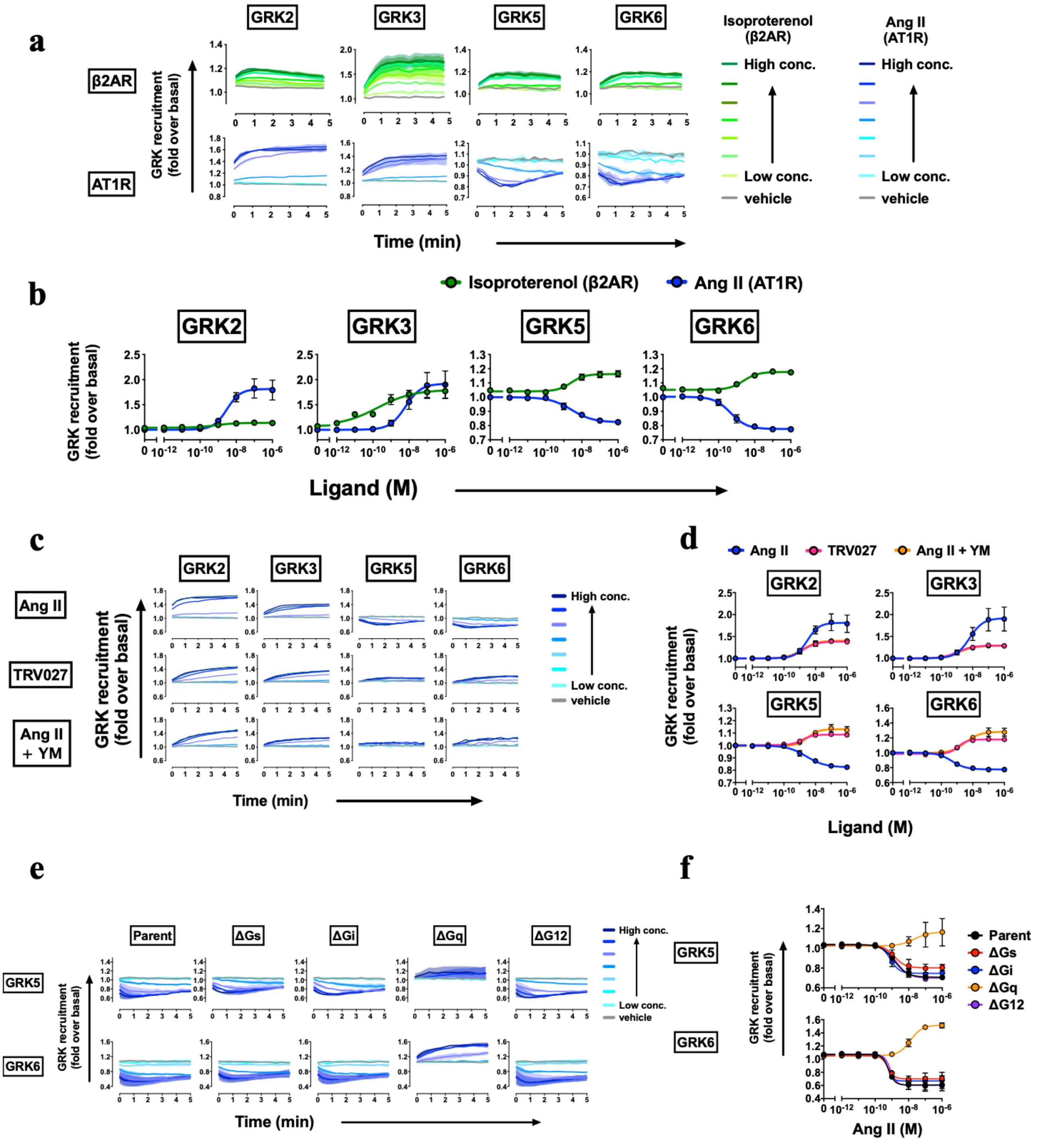
a
GRK2
GRK3
GRK5
GRK6
β2AR
AT1R
GRK recruitment (fold over basal)
Time (min)
Isoproterenol (β2AR)
Ang II (AT1R)
High conc.
Low conc.
vehicle
b
Isoproterenol (β2AR)
Ang II (AT1R)
Ligand (M)
c
Ang II
TRV027
Ang II + YM
d
e
Parent
ΔGs
ΔGi
ΔGq
ΔG12
f
Ang II (M)

Supplementary Fig. 15 GRK recruitment to AT1R under the Gq-inactivated condition.

a, b, Representative luminescent kinetics (a) and concentration-response curves (b) of the NanoBiT-GRK-recruitment assay. The parent cells individually expressing the LgBiT-fused GRK constructs along with AT1R-Sm or β2AR-Sm were stimulated with the corresponding GPCR ligands. Isoproterenol, a ligand for β2AR. **c-f,** Representative luminescent kinetics (c), luminescent kinetics (e) and concentration-response curves (d, f) of the NanoBiT-GRK-recruitment assay. The parent cells (c, d) and the G-protein-deficient cell lines (e, f) expressing the indicated GRK-Lg constructs along with AT1R-Sm were subjected to the NanoBiT assay using Ang II (with or without 1 μM YM-254890 pretreatment) or TRV027. In Fig. 15a, e, lines and shaded regions represent mean and SEM, respectively, of 3 (e) or 3-4 (a) independent experiments with each performed in duplicate. In Fig. 15b, d, f, symbols and error bars represent mean and SEM, respectively, of 3 (d, f) or 3-4 (b) independent experiments with each performed in duplicate.

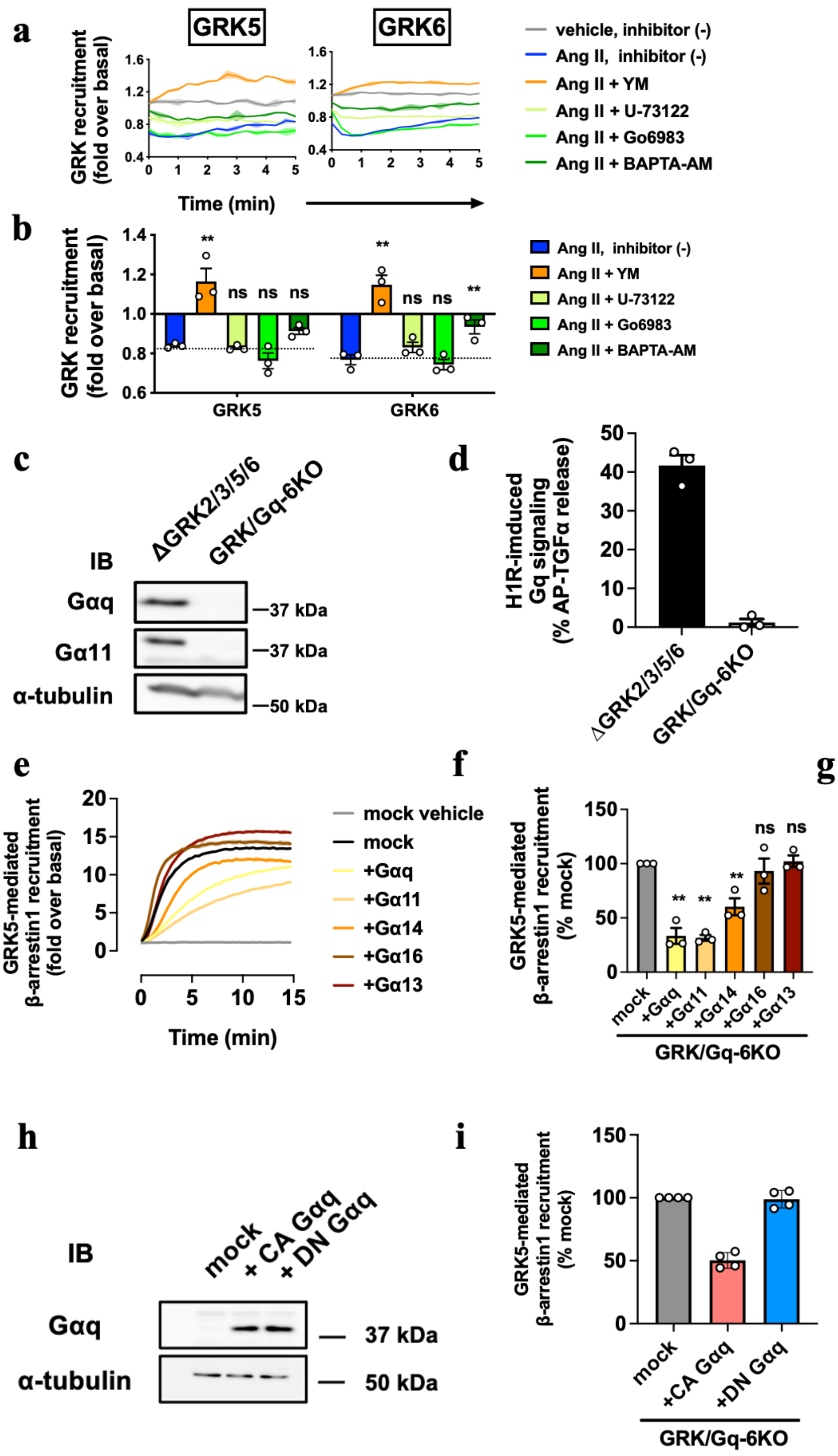
a
GRK5
GRK6
GRK recruitment (fold over basal)
Time (min)
vehicle, inhibitor (-)
Ang II, inhibitor (-)
Ang II + YM
Ang II + U-73122
Ang II + Go6983
Ang II + BAPTA-AM
b
GRK recruitment (fold over basal)
GRK5
GRK6
c
IB
ΔGRK2/3/5/6
GRK/Gq-6KO
Gαq
Gα11
α-tubulin
37 kDa
37 kDa
50 kDa
d
H1R-imduced Gq signaling (% AP-TGFα release)
e
GRK5-mediated β-arrestin1 recruitment (fold over basal)
Time (min)
mock vehicle
mock
+Gαq
+Gα11
+Gα14
+Gα16
+Gα13
f
GRK5-mediated β-arrestin1 recruitment (% mock)
GRK/Gq-6KO
g
H1R- induced Gq signaling (% AP-TGFα release)
GRK/Gq-6KO
h
IB
mock
+ CA Gαq
+ DN Gαq
Gαq
α-tubulin
37 kDa
50 kDa
i
GRK5-mediated β-arrestin1 recruitment (% mock)
mock
+CA Gαq
+DN Gαq
GRK/Gq-6KO

Supplementary Fig. 16 Effect of Gq downstream signaling on the GRK5/6 utilization.

a, b, Effect of Gq-signaling inhibitors. The parent cells expressing AT1R-Sm and GRK5-Lg were pretreated with the indicated inhibitors (1 μM YM-254890, 3 μM U-73122, 3 μM Go6983 and 10 μM BAPTA-AM) and stimulated with 1 μM Ang II. Data are shown as representative luminescent kinetics (a) and quantifications (b). **c, d,** Generation of the GRK/Gq-6KO cells. The *GNAQ* and the *GNA11* genes in ΔGRK2/3/5/6 (CL2) were mutated by the CRISPR-Cas9 system (Methods) and the resulting cells were referred to as the GRK/Gq-6KO cells. The lack of expression and function of Gq was validated by the western blot analysis (c) and the TGF-α shedding assay (d), respectively. For the TGF-α shedding assay, the Gq-coupled histamine H1 receptor was expressed along with the AP-TGFα reporter and histamine (10 μM)-induced AP-TGFα release response was measured. **e-g,** Effect of the individual Gq family members. GRK5-mediated β-arrestin1 recruitment response (e, f) as well as Gq signaling function (g) were assessed by the NanoBiT-β-arrestin recruitment assay and the TGF-α shedding assay, respectively. The indicated Gα subunits were individually expressed along with GRK5-FLAG, Lg-β-arrestin1 and AT1R-Sm and Ang II (1 μM)-induced β-arrestin1-recruitment response was measured. For simplicity, vehicle kinetics was shown only for the mock-transfected (i.e., no Gα transfection) cells. The TGFα shedding assay was performed as in (d). Note that the Gq family consists of the 4 members (Gαq, Gα11, Gα14 and Gα16) and that the Gα13 subunit was used as a negative control. **h, i,** A constitutively active mutant (Q209L, CA Gαq) or a dominant-negative mutant (Q209L/D277L, DN Gαq) was expressed along with GRK5-FLAG, Lg-β-arrestin1 and AT1R-Sm in GRK/Gq-6KO cells. Expression levels of the mutant Gαq (left panel) and GRK5-mediated β-arrestin1 recruitment response upon Ang II stimulation (right panel) were assessed by Western blot and the NanoBiT-β-arrestin recruitment assay, respectively. In Fig. 16a lines and shaded regions represent mean and SEM, respectively, of 3 independent experiments with each performed in duplicate. In Fig. 16d, f, g, i, bars and error bars represent mean and SEM, respectively, of 3 (b, f, g) or 4 (i) independent experiments with each performed in duplicate (b, f, i) or triplicate (g). In Fig. 16b, f, * and ** represent $P < 0.05$ and 0.01, respectively, with one-way (f) or two-way (b) ANOVA followed by the Dunnett's test for multiple comparison analysis with reference to the Ang II + inhibitor (-) (b) or the mock (f). ns, not significantly different between the groups. See Supplementary statistics data file for additional statistics and exact *P* values.

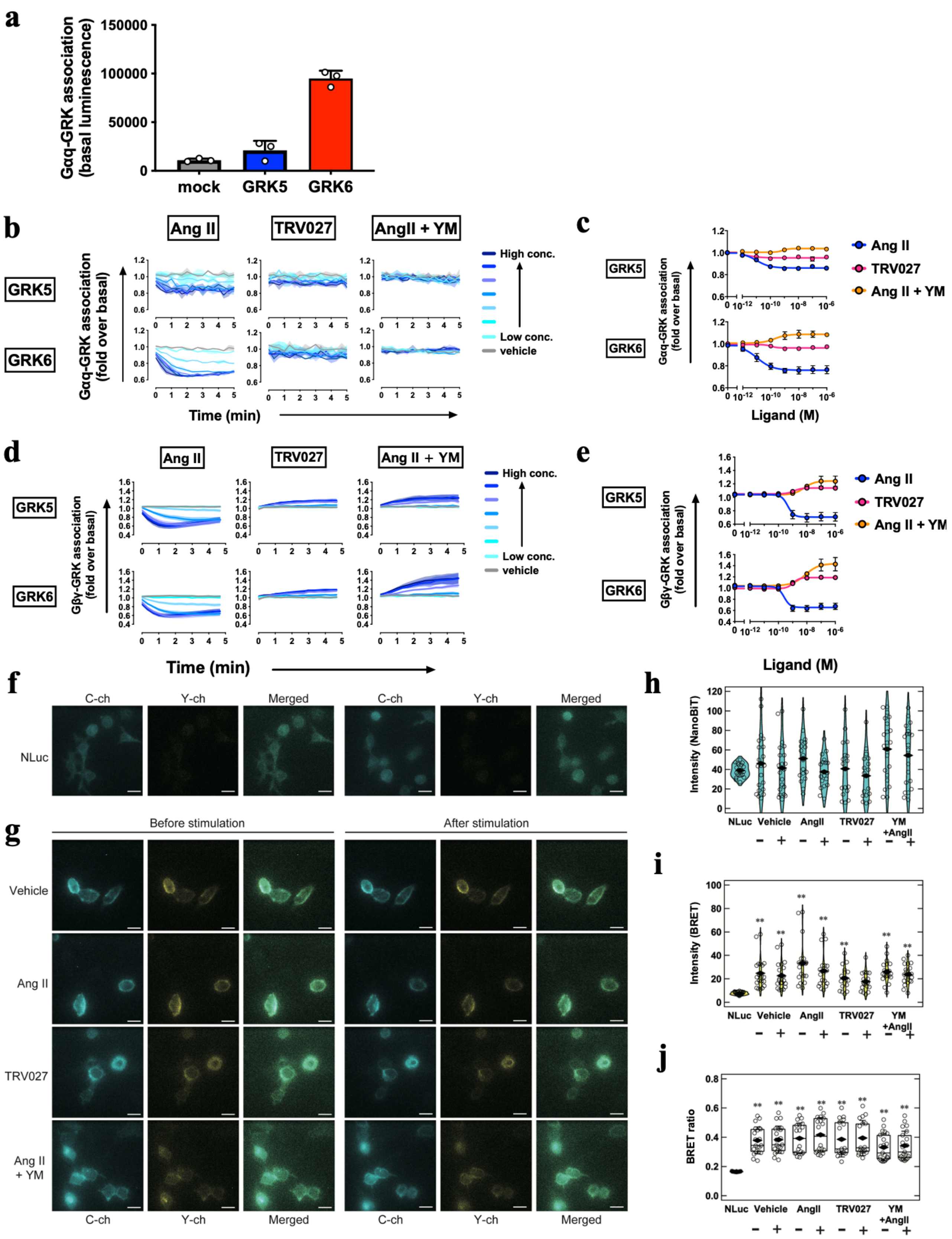
a
Gαq-GRK association (basal luminescence)
150000
100000
50000
0
mock
GRK5
GRK6
b
Ang II
TRV027
AngII + YM
GRK5
GRK6
Gαq-GRK association (fold over basal)
High conc.
Low conc.
vehicle
Time (min)
c
GRK5
GRK6
Gαq-GRK association (fold over basal)
Ang II
TRV027
Ang II + YM
Ligand (M)
d
Ang II
TRV027
Ang II + YM
GRK5
GRK6
Gβγ-GRK association (fold over basal)
High conc.
Low conc.
vehicle
Time (min)
e
GRK5
GRK6
Gβγ-GRK association (fold over basal)
Ang II
TRV027
Ang II + YM
Ligand (M)
f
C-ch
Y-ch
Merged
NLuc
g
Before stimulation
After stimulation
Vehicle
Ang II
TRV027
Ang II + YM
h
Intensity (NanoBiT)
NLuc
Vehicle
AngII
TRV027
YM +AngII
i
Intensity (BRET)
j
BRET ratio

Supplementary Fig. 17 GRK5/6 association to Gq and conformational change under the Gq-inactivated condition.

a, Basal complex levels of the Gq-GRK complex. The parent cells expressing the indicated Sm-GRK constructs along with the Gαq-Lg, the untagged Gβ1, the untagged Gγ2 subunits and AT1R were loaded with coelenterazine, a substrate for the NanoBiT luciferase, and baseline luminescent signals were measured. **b, c,** Representative luminescent kinetics (b) and concentration-response curves (c) of the NanoBiT-Gαq-GRK proximity assay. The parent cells in (a) were subjected to the NanoBiT assay using Ang II (with or without 1 μM YM-254890) and TRV027. **d, e,** Representative luminescent kinetics (d) and concentration-response curves (e) of the NanoBiT-Gβγ-GRK proximity assay. Same as in (b, c), but using a combination of GRK-Lg, the Sm-Gβ1 and the Gγ2 subunits and AT1R. **f-j,** NanoBiT-BRET imaging. The parent cells expressing Nanoluc (Nluc) were evaluated for their bleed-through from the C-channel (425-to-475-nm band path filter) to the Y-channel (515-to-550-nm band path filter), corresponding to the NanoBiT-derived luminescent signal and the mVenus-mediated BRET signal, respectively (f). The parent cells expressing Gαq-Lg, Sm-GRK6 and AT1R-mVenus were loaded with furimazine. Luminescent signals (NanoBiT and BRET) upon the indicated conditions (Vehicle, 1 μM Ang II, 1 μM TRV027 or 1 μM Ang II pretreated with 1 μM YM-254890) were imaged via the C-channel and the Y-channel, respectively (g). Note that the enlarged microscopy images under the same conditions were shown in Figure 6i. Luminescence intensity from complemented NanoBiT (h), Fluorescence intensity from the BRET index (i) and the BRET ratio (j) under indicated condition (- before stimulation, + after stimulation). In Fig. 17b, d, lines and shaded regions represent mean and SEM, respectively, of 3 independent experiments with each performed in duplicate. In Fig. 17a, bars and error bars represent mean and SEM, respectively, of 3 independent experiments with each performed in duplicate. In Fig. 17c, e, symbols and error bars represent mean and SEM, respectively, of 3 independent experiments with each performed in duplicate. In Fig. 17h, i, shaded regions represent histogram, of 17-21 cells. In Fig. 17j, boxes, filled squares, center lines and whiskers represents interquartile range, mean, median, and maxima and minima, respectively. In Fig. 17h, i, j, * and ** represent $P < 0.05$ and 0.01, respectively, with one-way ANOVA followed by the Dunnett's test for multiple comparison analysis with reference to the Nluc. ns, not significantly different between the groups. See Supplementary statistics data file for additional statistics and exact *P* values.

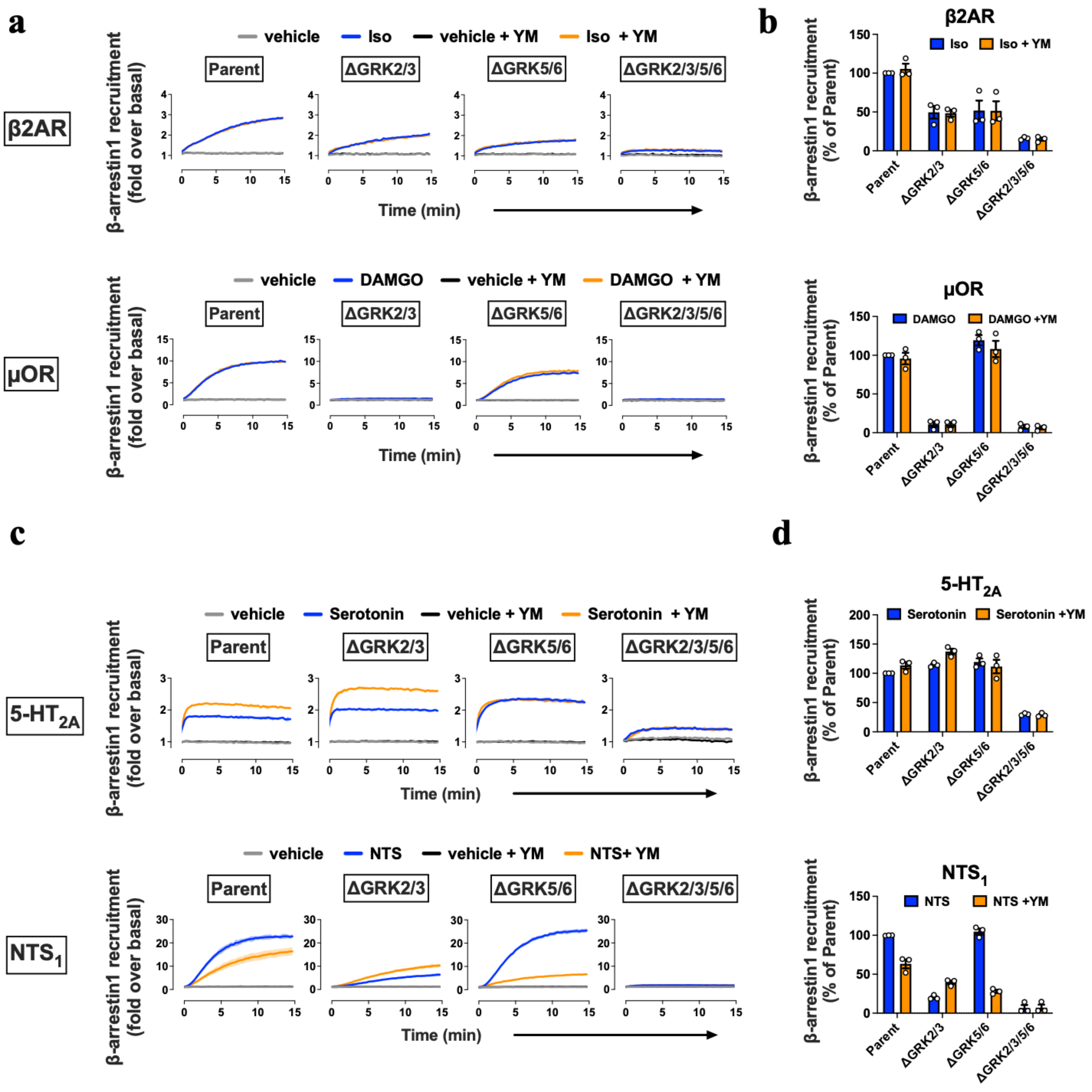

Supplementary Fig. 18 YM-triggered GRK-subtype selectivity switch in Gq- and non-Gq-coupled receptors. **a, b,** Non-Gq-coupled receptors. The Gs-coupled β2AR and the Gi-coupled μOR both containing the C-terminal SmBiT were expressed with the Lg-β-arrestin1 in the parent and the GRK-deficient cell lines. Representative luminescent kinetics (a) and concentration-response curves (b) upon stimulated with 10 μM isoproterenol (β2AR) and 10 μM DAMGO (μOR) in the presence or absence of 1 μM YM-254890 were shown. **c, d,** Gq-coupled receptors. Same as in (a, b), but using the Gq-coupled 5-HT_{2A} and the Gq-coupled NTS_1 receptors and corresponding ligands (10 μM serotonin and 1 μM neurotensin, respectively). In Fig. 18a, c, lines and shaded regions represent mean and SEM, respectively, of 3 independent experiments with each performed in duplicate. In Fig. 18b, d, bars and error bars represent mean and SEM, respectively, of 3 independent experiments with each performed in duplicate.

Uncropped images of Western blot analysis

Supplementary Fig. 2e

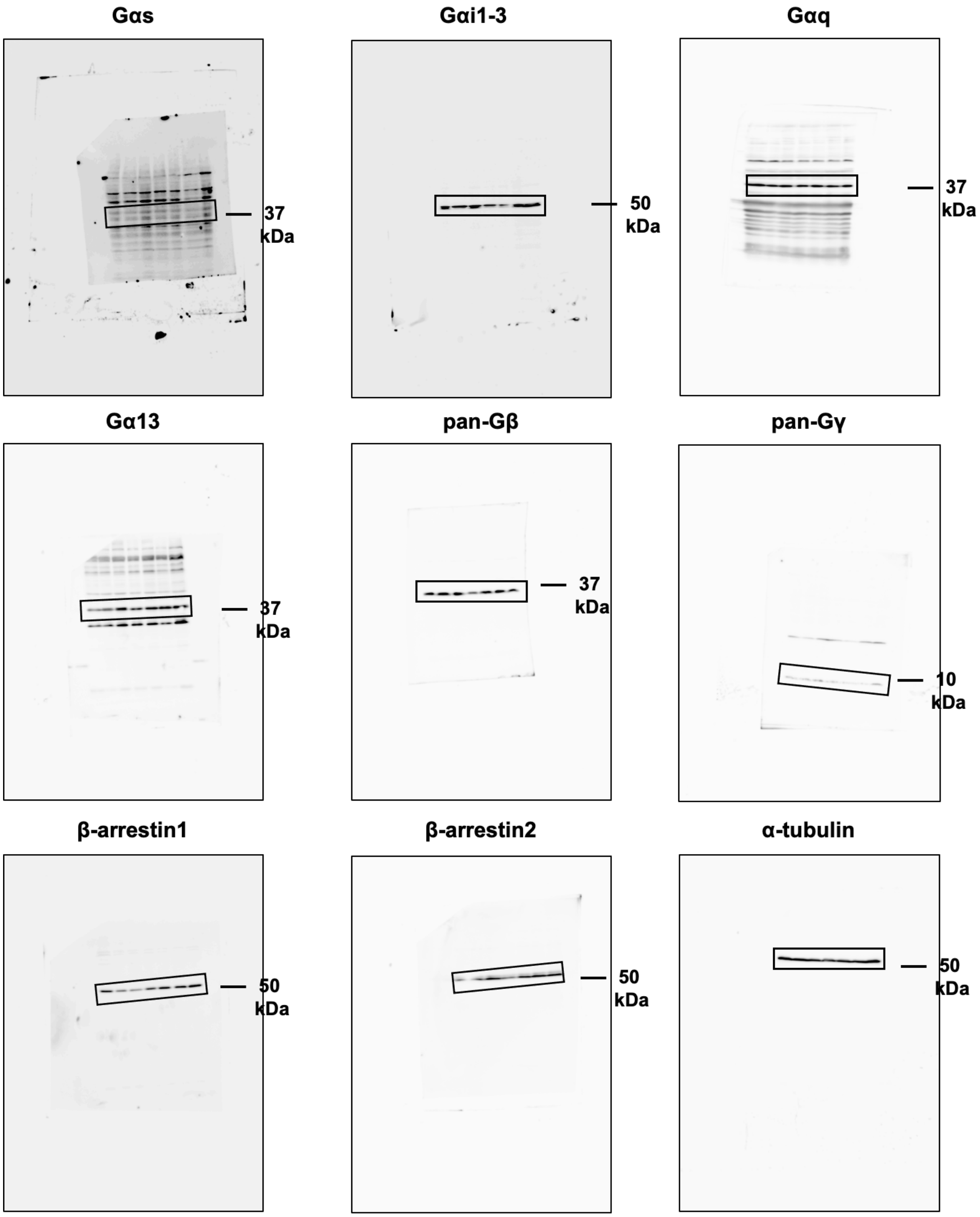

Supplementary Fig. 4c

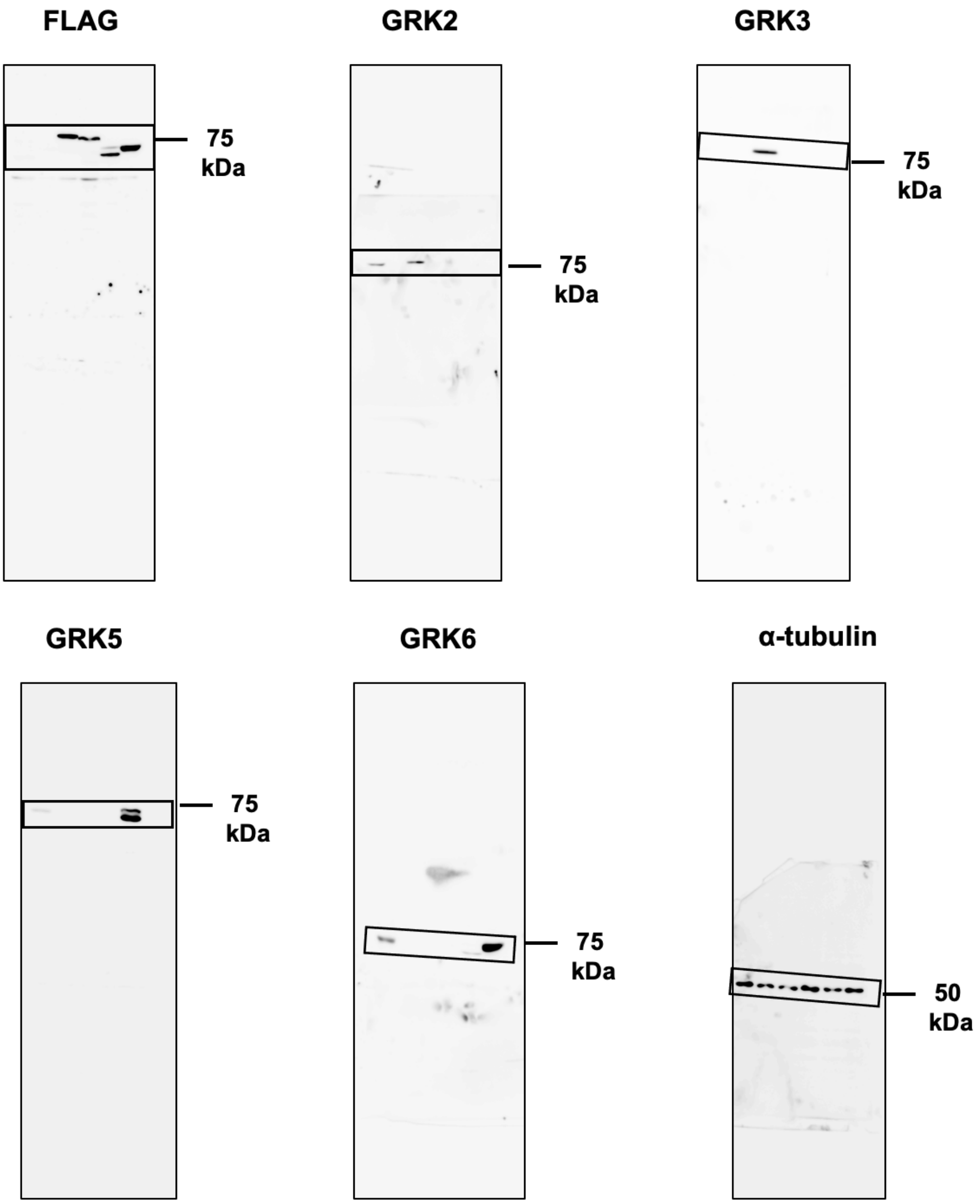

Supplementary Fig. 5a

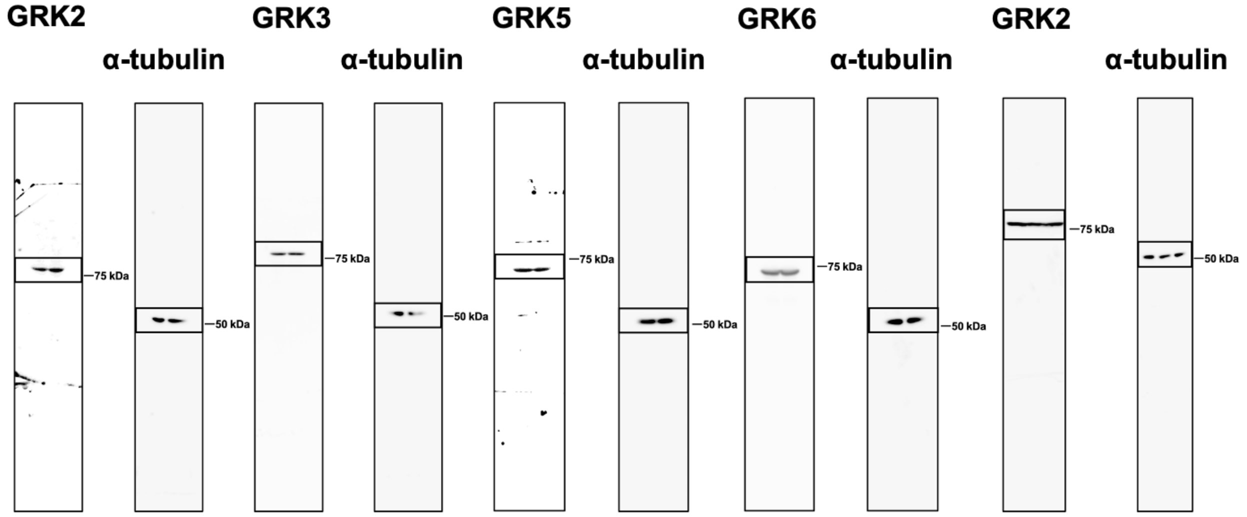

Supplementary Fig. 8a

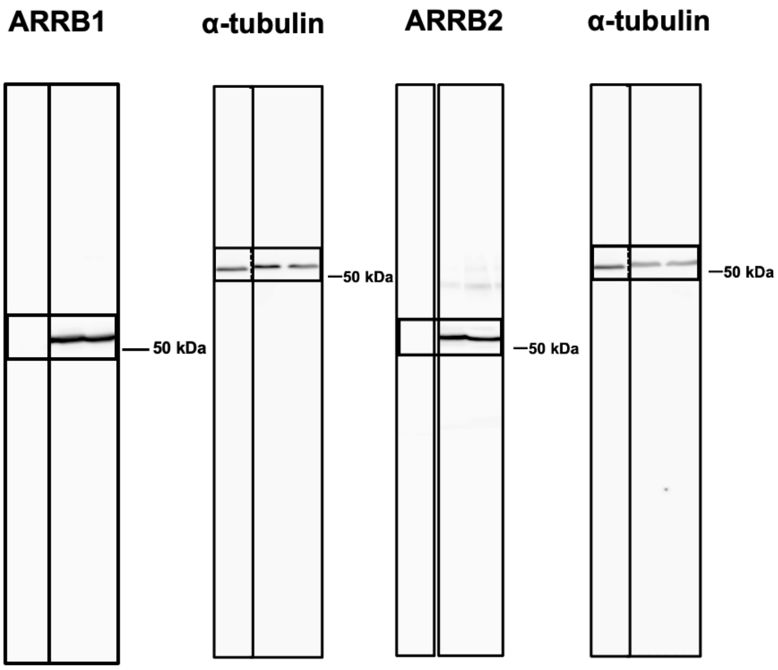

Supplementary Fig. 16c

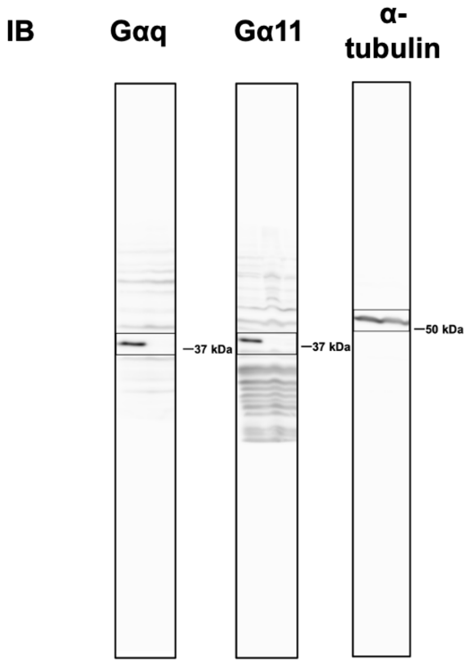

Supplementary Fig. 16h

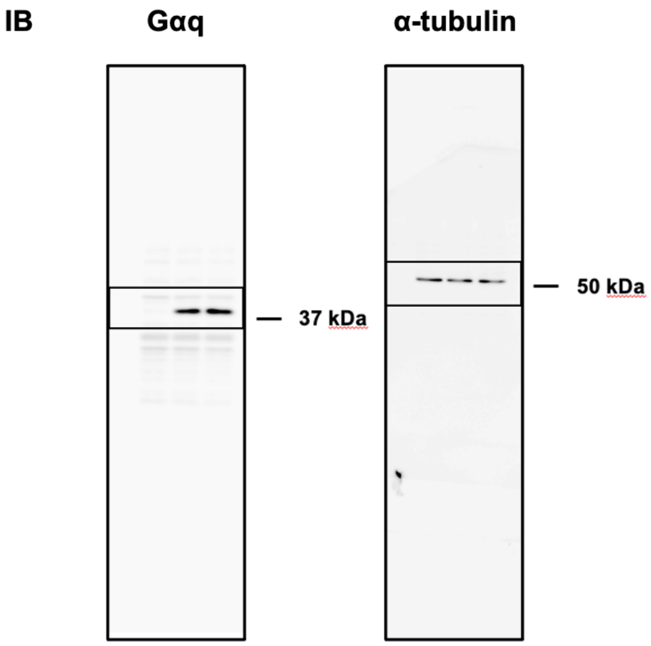

Supplement: Supplementary file 1 — Supplementary Information [file 41467_2022_28056_MOESM1_ESM.pdf]
