# Supplementary material for: Heterotrimeric Gq proteins act as a switch for GRK5/6 selectivity underlying β-arrestin transducer bias
Source: Nat Commun. 2022 Jan 25;13:487. doi: 10.1038/s41467-022-28056-7 (PMC8789823; doi:10.1038/s41467-022-28056-7)
Supplement: Supplementary file 3 — Description of Additional Supplementary Files [file 41467_2022_28056_MOESM3_ESM.pdf]

Description of Additional Supplementary Files

File name: Supplementary Movie 1

Description: Two-color SMT analysis of AT1R and GRK5

File name: Supplementary Movie 2

Description: Two-color SMT analysis of AT1R and GRK2

File name: Supplementary Movie 3

Description: NanoBiT-BRET imaging of AT1-Gq-GRK assembly
