# Supplementary material for: Heterotrimeric Gq proteins act as a switch for GRK5/6 selectivity underlying β-arrestin transducer bias
Source: Nat Commun. 2022 Jan 25;13:487. doi: 10.1038/s41467-022-28056-7 (PMC8789823; doi:10.1038/s41467-022-28056-7)
Supplement: Supplementary file 7 — Reporting Summary [file 41467_2022_28056_MOESM7_ESM.pdf]

nature portfolio

Corresponding author(s): Yasushi Sako and Asuka Inoue

Last updated by author(s): Dec 10, 2021

Reporting Summary

Nature Portfolio wishes to improve the reproducibility of the work that we publish. This form provides structure for consistency and transparency in reporting. For further information on Nature Portfolio policies, see our Editorial Policies and the Editorial Policy Checklist.

Statistics

For all statistical analyses, confirm that the following items are present in the figure legend, table legend, main text, or Methods section.

n/a	Confirmed	
☐	☒	The exact sample size (*n*) for each experimental group/condition, given as a discrete number and unit of measurement
☐	☒	A statement on whether measurements were taken from distinct samples or whether the same sample was measured repeatedly
☐	☒	The statistical test(s) used AND whether they are one- or two-sided<br>*Only common tests should be described solely by name; describe more complex techniques in the Methods section.*
☒	☐	A description of all covariates tested
☐	☒	A description of any assumptions or corrections, such as tests of normality and adjustment for multiple comparisons
☐	☒	A full description of the statistical parameters including central tendency (e.g. means) or other basic estimates (e.g. regression coefficient) AND variation (e.g. standard deviation) or associated estimates of uncertainty (e.g. confidence intervals)
☐	☒	For null hypothesis testing, the test statistic (e.g. *F*, *t*, *r*) with confidence intervals, effect sizes, degrees of freedom and *P* value noted<br>*Give P values as exact values whenever suitable.*
☐	☒	For Bayesian analysis, information on the choice of priors and Markov chain Monte Carlo settings
☒	☐	For hierarchical and complex designs, identification of the appropriate level for tests and full reporting of outcomes
☒	☐	Estimates of effect sizes (e.g. Cohen's *d*, Pearson's *r*), indicating how they were calculated

Our web collection on statistics for biologists contains articles on many of the points above.

Software and code

Policy information about availability of computer code

Data collection	SoftMax Pro Version7.0.3, AIS, Metamorph Version7.10
Data analysis	The following software or algolism were used inthis study: GraphPad Prism 8, FlowJo software, ImageJ, AAS; LabVIEW, Igor Pro 8, Optimized CRISPR design(https://zlab.bio/guide-design-resources). The SMT analysis method (smDynamicsAnalyzer: https://github.com/masataka-yanagawa/IgorPro8-smDynamicsAnalyzer) and the image processing method for SMT and NanoBiT-BRET experiments (https://github.com/masataka-yanagawa/ImageJ-macro-ImageProcessingSMT) are available.

For manuscripts utilizing custom algorithms or software that are central to the research but not yet described in published literature, software must be made available to editors and reviewers. We strongly encourage code deposition in a community repository (e.g. GitHub). See the Nature Portfolio guidelines for submitting code & software for further information.

Data

Policy information about availability of data

All manuscripts must include a data availability statement. This statement should provide the following information, where applicable:

- Accession codes, unique identifiers, or web links for publicly available datasets
- A description of any restrictions on data availability
- For clinical datasets or third party data, please ensure that the statement adheres to our policy

All data generated or analyzed during this study are included in this article, its Supplementary Information and Source Data file or from the corresponding authors on reasonable request. the Source data of Fig.1-6 and Supplementary Fig.2-18 are provided in the separated Source Data file.

Field-specific reporting

Please select the one below that is the best fit for your research. If you are not sure, read the appropriate sections before making your selection.

☒ Life sciences ☐ Behavioural & social sciences ☐ Ecological, evolutionary & environmental sciences

For a reference copy of the document with all sections, see nature.com/documents/nr-reporting-summary-flat.pdf

Life sciences study design

All studies must disclose on these points even when the disclosure is negative.

Sample size	No statistical method was used to determine the sample size. We performed three or more independent experiments or evaluated twenty or more cells in NanoBiT-based GPCR assay or single molecule tracking analysis, respectively, based on other studies with similar methodology (PMID: 31160049, 30228224)
Data exclusions	No data was systematically excluded.
Replication	In vitro experiments were independently performed two times for single-molecule tracking microscopy and at least three times for all of the other experiments. All attempts at replication were succeeded.
Randomization	No randomization was attempted or needed. This was not a clinical trial or animal study that is dependent on randomization. All the experiments were performed using immortalized cell lines ans all variables could be controlled.
Blinding	No blinding was attempted or needed. There was no group allocation performed in this study.

Reporting for specific materials, systems and methods

We require information from authors about some types of materials, experimental systems and methods used in many studies. Here, indicate whether each material, system or method listed is relevant to your study. If you are not sure if a list item applies to your research, read the appropriate section before selecting a response.

Materials & experimental systems

n/a	Involved in the study	
☐	☒	Antibodies
☐	☒	Eukaryotic cell lines
☒	☐	Palaeontology and archaeology
☒	☐	Animals and other organisms
☒	☐	Human research participants
☒	☐	Clinical data
☒	☐	Dual use research of concern

Methods

n/a	Involved in the study	
☒	☐	ChIP-seq
☐	☒	Flow cytometry
☒	☐	MRI-based neuroimaging

Antibodies

Antibodies used

Primary antibodies used in this study:
anti-GRK2 rabbit monoclonal antibody (R&D, MAB43391, 2089B, lot CKLB021901A, 1:2000 dilution)
anti-GRK3 rabbit monoclonal antibody (CST, #80362, D8G6V, lot 80362S, 1:1000 dilution)
anti-GRK5 mouse monoclonal antibody (Santa Cruz Biotechnologies, sc-518005, D-9, lot J3117, 1: 5000 dilution)
anti-GRK6 rabbit monoclonal antibody (CST, #5878, D1A4, lot 5878S, 1:1000 dilution)
anti-α-tubulin mouse monoclonal antibody (Santa Cruz Biotechnologies, sc-32293, DM1A, lot K1414, 1:2000 dilution)
anti-Flag-epitope tag mouse monoclonal antibody (Wako Pure Chemicals, 014-22383, Clone 1E6, 1:1000 dilution)
anti-Gs/olf mouse monoclonal antibody (Santa Cruz Biotechnologies, sc-55546, E-7, lot B2307, 1:1000 dilution)
anti-Gi mouse monoclonal antibody (Neweast biosciences, 26003, lot. PH063,unknown clone: http://www.neweastbio.com/AntiGTPase/23, 1:1000 dilution)
anti-Gq goat poyuclonal antibody (abcam, ab128060, D-6, lot GR108939-6, 1:2000 dilution)
anti-G11 mouse monoclonal antibody (Santa Cruz Biotechnologies, sc-390382, D-6, lot D0113, 1:2000 dilution)
anti-G13 rabbit monoclonal antibody (abcam, ab128900, EPR5436, GR90067-7, 1:1000 dilution)
anti-Gbeta mouse monoclonal antibody (Santa Cruz Biotechnologies, sc-166123, H-1, lot F2718, 1:1000 dilution)
anti-Ggamma mouse monoclonal antibody (Santa Cruz Biotechnologies, sc-166419, C-5, lot E1517, 1:1000 dilution)
anti-beta arrestin1 rabbit monoclonal antibody (CST, #12697, D8O3J, lot 1, 1:1000 dilution)
anti-beta arrestin2 rabbit monoclonal antibody (CST, #3857, C16D9, lot 2, 1:1000 dilution)
anti-P-p44/42 MAPK(T202/Y204) rabbit monoclonal antibody (CST, #8544, D13.14.4E, lot 3, 1:10000 dilution)
and anti-p44/42 MAPK rabbit monoclonal antibody (CST, #4695, 137F5, lot 2, 1:10000 dilution)

Secondary antibodies that were conjugated with horseradish peroxidase (HRP) used in this study:

	Anti-mouse IgG, HRP-Linked F(ab')2 Fragment Sheep (GE Healthcare, NA9310, lot F17365253)<br>Anti-Rabbit IgG, HRP-Linked F(ab')2 Fragment Donkey (GE Healthcare, NA9340, lot 17041889)<br>and anti-goat IgG (American Qualex, A201PS lot 7A0327H).
Validation	All purchased antibodies were well validated by the manufactures in their specific data sheets as follows. In Anti-GRK antibodies were additionally knockout validated in this manuscript.<br>anti-GRK2 rabbit monoclonal antibody: https://www.rndsystems.com/products/human-mouse-rat-grk2-antibody-2089b_mab43391<br>anti-GRK3 rabbit monoclonal antibody: https://www.cellsignal.jp/products/primary-antibodies/grk3-d8g6v-rabbit-mab/80362<br>anti-GRK5 mouse monoclonal antibody: https://www.scbt.com/p/grk-5-antibody-d-9<br>anti-GRK6 rabbit monoclonal antibody: https://www.cellsignal.jp/products/primary-antibodies/grk6-d1a4-rabbit-mab/5878<br>anti-α-tubulin mouse monoclonal antibody:https://www.scbt.com/ja/p/alpha-tubulin-antibody-dm1a<br>anti-Flag-epitope tag mouse monoclonal antibody: https://labchem-wako.fujifilm.com/jp/product/detail/W01W0101-2238<br>anti-Gs/olf mouse monoclonal antibody:https://www.scbt.com/ja/p/galpha-s-olf-antibody-e-7<br>anti-Gi mouse monoclonal antibody: http://www.neweastbio.com/AntiGTPase/23<br>anti-Gq goat poyuclonal antibody: https://www.abcam.co.jp/gnaq-antibody-ab128060<br>anti-G11 mouse monoclonal antibody: https://www.scbt.com/p/galpha-11-antibody-d-6<br>anti-G13 rabbit monoclonal antibody: https://www.abcam.co.jp/gna13-antibody-epr5436-ab128900<br>anti-Gbeta mouse monoclonal antibody: https://www.scbt.com/ja/p/gbeta-antibody-h-1<br>anti-Ggamma mouse monoclonal antibody: https://www.scbt.com/ja/p/ggamma-2-3-4-7-antibody-c-5<br>anti-beta arrestin1 rabbit monoclonal antibody: https://en.cellsignal.jp/products/primary-antibodies/b-arrestin-1-d8o3j-rabbit-mab/12697<br>anti-beta arrestin2 rabbit monoclonal antibody: https://en.cellsignal.jp/products/primary-antibodies/b-arrestin-2-c16d9-rabbit-mab/3857<br>anti-P-p44/42 MAPK(T202/Y204) rabbit monoclonal antibody: https://en.cellsignal.jp/products/antibody-conjugates/phospho-p44-42-mapk-erk1-2-thr202-tyr204-d13-14-4e-xp-rabbit-mab-hrp-conjugate/8544<br>anti-p44/42 MAPK rabbit monoclonal antibody: https://en.cellsignal.jp/products/primary-antibodies/p44-42-mapk-erk1-2-137f5-rabbit-mab/4695<br>Anti-mouse IgG: https://www.cytivalifesciences.co.jp/catalog/0428<br>Anti-Rabbit IgG: https://www.cytivalifesciences.co.jp/catalog/0428<br>and anti-goat IgG: manufacture's data sheet is currently unavailable. This antibody was used in several papers from other groups (PMID: 10880057,32816022)

Eukaryotic cell lines

Policy information about cell lines

Cell line source(s)	HEK293A cells were from Thermo Fisher Scientific, Cat R70507
Authentication	Cell lines are maintained by the supplier. No additional authentication was performed by the authors of this study.
Mycoplasma contamination	All Cell lines are ensured the absence of mycoplasma contamination using MycoAlert Mycoplasma detection kit (Lonza).
Commonly misidentified lines<br>(See ICLAC register)	None used.

Flow Cytometry

Plots

Confirm that:

☒ The axis labels state the marker and fluorochrome used (e.g. CD4-FITC).

☒ The axis scales are clearly visible. Include numbers along axes only for bottom left plot of group (a 'group' is an analysis of identical markers).

☒ All plots are contour plots with outliers or pseudocolor plots.

☒ A numerical value for number of cells or percentage (with statistics) is provided.

Methodology

Sample preparation	HEK293 cells were transfected by combining 2.5 μl of the polyethylenimine solution (1 mg mL-1) and 200 ng of a plasmid encoding FLAG epitope-tagged GPCR (volumes denote per well in a 6-well plate).
Instrument	EC800 flow cytometer (Sony)
Software	FlowJo software (FlowJo)
Cell population abundance	N/A (since we analysed a single cell type and obtained data from all observed cells.)
Gating strategy	N/A (since we used all of the recorded fluorescent signals and calculated a simple mean value, description of gating strategy or the plot representation was not performed.

☒ Tick this box to confirm that a figure exemplifying the gating strategy is provided in the Supplementary Information.
